# Antimicrobial Activity of Gemini Surfactants with Azapolymethylene Spacer

**DOI:** 10.3390/molecules25184054

**Published:** 2020-09-04

**Authors:** Iwona Kowalczyk, Marta Pakiet, Adrianna Szulc, Anna Koziróg

**Affiliations:** 1 Department of Bioactive Products, Faculty of Chemistry, Adam Mickiewicz University Poznan, 61-614 Poznan, Poland; mpakiet@amu.edu.pl (M.P.); adaszulc@amu.edu.pl (A.S.); 2 Institute of Fermentation Technology and Microbiology, Faculty of Biotechnology and Food Science, Lodz University of Technology, 90-924 Lodz, Poland; anna.kozirog@p.lodz.pl

**Keywords:** gemini surfactants, antimicrobial activity, minimal inhibitory concentration, critical micelle concentration

## Abstract

A series of 21 azapolymethylene gemini surfactants were obtained. The synthesis of the title surfactants in one- or two-step reaction proceeds with good yields. The structure and the purity of the synthesized compounds were determined by ^1^H and ^13^C NMR, ESI-MS spectra, and elemental analysis. Moreover, 2D COSY, HMBC, and HSQC spectra were performed. The minimal inhibitory concentrations (MIC) of the synthesized compounds were determined against fungi: *Candida albicans, Aspergillus niger*, *Penicillium chrysogenum* and bacteria: *Escherichia coli,* *Pseudomonas aeruginosa, Staphylococcus aureus,* and *Bacillus subtilis*. Also, the critical micelle concentrations (CMC) were determined. The relationship between antimicrobial and surface activity and surfactant structure has been determined.

## 1. Introduction

Quaternary alkylammonium salts are amphiphilic compounds which consist of hydrophobic and hydrophilic parts. The hydrophilic part constitutes a quaternary nitrogen atom whereas the hydrophobic part is the alkyl chains of different lengths. Two quaternary ammonium compounds are linked by a spacer at polar heads or close to them form gemini surfactants [1,2,3,4,5]. The surface tension and critical micelle concentration of gemini surfactants, as well as their antimicrobial activity, expressed by minimal inhibitory concentration, are significantly lower, even hundreds of times, in comparison to their monomeric analogs [6,7,8,9,10,11,12,13]. This is of great ecological importance because to achieve a similar surface or antimicrobial effect, much smaller amounts of the substance can be used.

In dilute solutions, surfactants exist as single molecules, monomers [1]. As the concentration increases, the surfactants associate to form micelles. The solution passes from the real solution to the colloidal one [14,15,16]. The concentration of the surfactant, in which the physical properties of the solution change rapidly due to the formation of associates, is called the critical micelle concentration (CMC). In concentrated solutions the surfactant above CMC is thermodynamically stable, remaining in the equilibrium with monomers [10,13,17,18].

The hydrocarbon chains, in an aqueous environment, form the core of the micelle while the hydrophilic groups are located outside of the micelle [19]. Therefore, the surfactant structure plays a key role in the formation of micelles [20,21]. 

Besides the excellent surface activity, gemini surfactants act also as a very efficient microbiocide and corrosion inhibitors, which is directly related to the adsorption capacity of cationic salts on negatively charged surfaces [22,23,24,25,26,27,28,29,30,31]. Adsorption of alkylammonium salt on the cell wall of the microorganism causes damage to the wall, and leakage of potassium ions and low molecular components of the bacterial cell, which in turn leads to cell death [32,33,34,35].

Due to the constantly growing demand for gemini surfactants as microbiocides, we focused on the synthesis of dimeric alkylammonium salts with azapolymethylene spacers and determination of the relationship between the structure of surfactants and their antimicrobial activity against bacteria and fungi. In this study, the critical micelle concentration of obtained compounds was also investigated.

## 2. Results and Discussion

### 2.1. Synthesis and Spectroscopic Characterisation of Gemini Surfactants

**Scheme 1:**
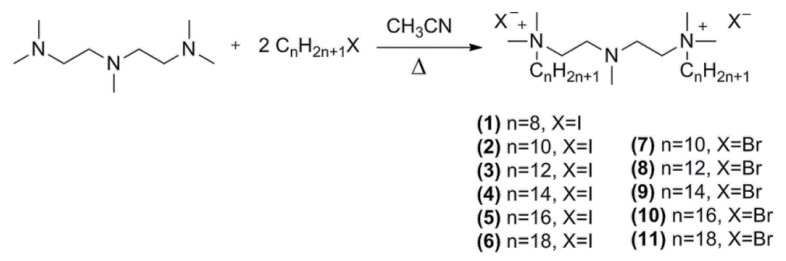
Synthesis of 3-methylaza-1,5-pentane-bis(*N*,*N*-dimethyl-*N*-alkylammonium) diiodides (compounds **1**–**6**) and dibromides (compounds **7**–**11**).

The 3-methylaza-1,5-pentane-bis(*N*,*N*-dimethyl-*N*-alkylammonium) diiodides (compounds **1**–**6**) and dibromides (compounds **7**–**11**) were obtained by the alkylation of a tertiary diamine with *n*–iodoalkanes or *n*–bromoalkanes. The synthesized group of surfactants contain a modified hydrophobic part and a counterion in the form of iodide or bromide (Scheme 1).

**Scheme 2:**
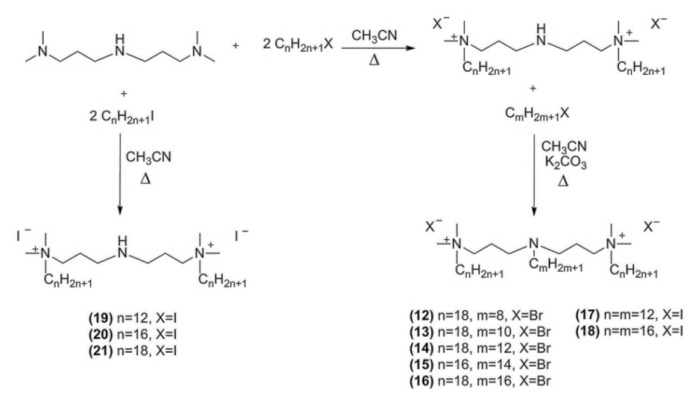
Synthesis of 4-aza-1,7-heptane-bis(*N*,*N*-dimethyl-*N*-alkylammonium) diiodides (**19**–**21**); 4-alkylaza-1,7-heptane-bis(*N*,*N*-dimethyl-*N*-alkylammonium) dibromides (**12**–**16**) and diiodides (**17**–**18**).

The other group of gemini surfactants with a secondary nitrogen atom in the spacer (Scheme 2) were prepared by alkylation of *N*,*N*,*N*′,*N*′-tetramethyl-1,4,7-triazaheptane with iodoalkanes in acetonitrile (compound **19**–**21**). Further alkylation in acetonitrile in the presence of potassium carbonate leads to gemini surfactants with an alkylated nitrogen atom in the spacer (compound **12**–**18**). The details of synthetic procedures of all obtained products are given in the Supplementary Material.

The structure of all synthesized compounds was determined by ^1^H and ^13^C NMR, ESI-MS spectra, or elemental analysis. Moreover, 2D COSY (Correlation Spectroscopy), HMBC (Heteronuclear Multiple Bond Correlation), and HSQC (Heteronuclear Single Quantum Correlation) spectra were performed for compound (**1**) and (**8**).

**Figure 1:**
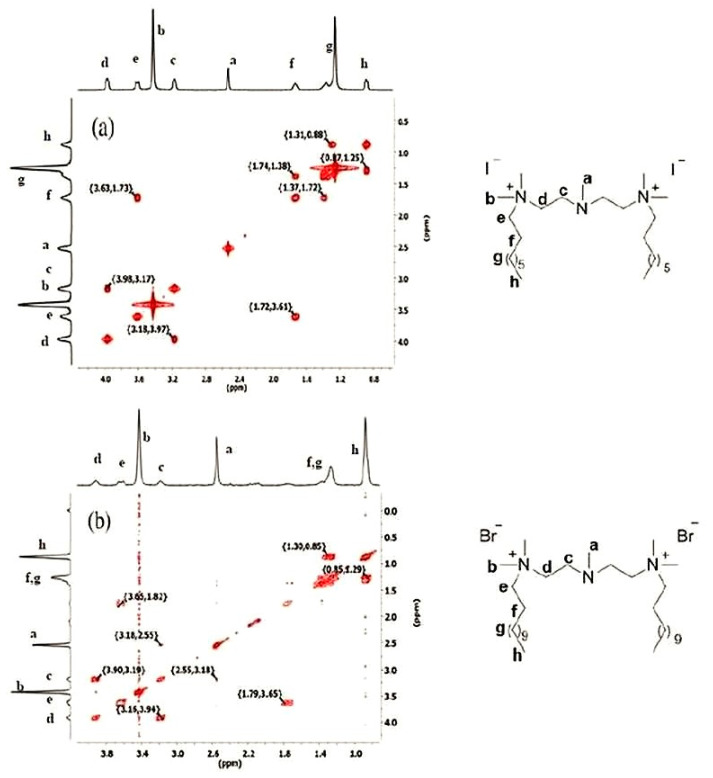
Spin–spin couplings obtained from ^1^H–^1^H COSY spectra of (**a**) compound (**1**) and (**b**) compound (**8**).

The ^1^H NMR spectra of compounds (**1**–**21**) show singlets in the range 3.43–3.19 ppm assigned to the N^+^–(CH_3_)_2_ protons. The characteristic singlet of N–CH_3_ of compound (**1**–**11**) were observed in the range 2.55–2.53 ppm. Proton singlets at 0.88–0.86 ppm were assigned to CH_3_(h). The other multiplets ranging from 1.76–1.25 ppm were assigned to methylene groups of the alkyl chain. These assignments are confirmed by ^1^H–^1^H COSY, which gives the relationship between protons in the molecule. The ^1^H–^1^H COSY spectra of compound (**8**) confirm the structure of the compound (Figure 1).

**Figure 2:**
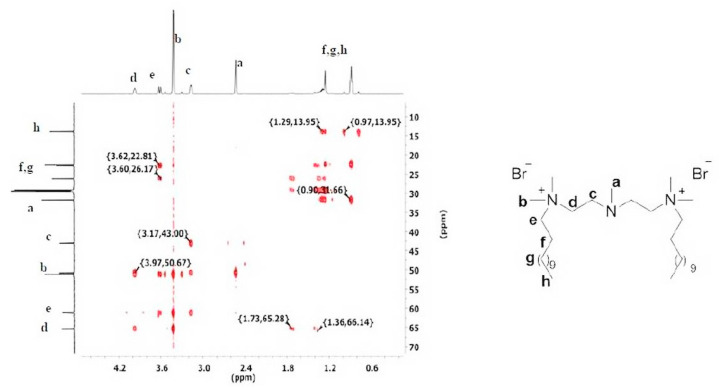
The ^1^H–^13^C HMBC spectra of compound (**8**).

The ^1^H–^13^C HMBC provides additional information on the structure of compound (**8**) (Figure 2). Protons (a) correlated with the carbon atoms (b,c), but they did not correlate with the carbon atoms (f,g,h). It confirms that the alkyl chains are linked to the side nitrogen atoms in the synthesized molecule.

Chemical shifts of the carbon atoms indicated the occurrence of a relationship between the length of the alkyl chain and the chemical shifts from the carbon atoms located in the vicinity to the quaternary nitrogen atom. Two important signals for the N^+^–CH_3_ group and N–CH_3_ were present at 51.57–51.52 ppm and 43.15–43.05 ppm, respectively. The spectra show characteristic signals at 14.01–13.94 ppm, which are assigned to CH_3_. The details of the structural analysis of all obtained products are given in the Supplementary Material.

### 2.2. MIC Study

Minimal inhibitory concentrations (MIC), i.e., the minimum concentration of the microbiocide at which further growth of tested microorganisms is stopped, have been determined for all azapolymethylene gemini surfactants against bacteria: *E. coli*, *P. aeruginosa*, *S. aureus*, and *B. subtilis* as well as microscopic fungi: *C. albicans*, *A. niger*, and *P. chrysogenum*.

The MIC values of 3-methylaza-1,5-pentane-bis(*N*,*N*-dimethyl-*N*-alkylammonium) diiodides (**1**–**6**) were shown in Table 1 and of 3-methylaza-1,5-pentane-bis(*N*,*N*-dimethyl-*N*-alkylammonium) dibromides (**7**–**11**) were shown in Table 2. 

**Figure 3:**
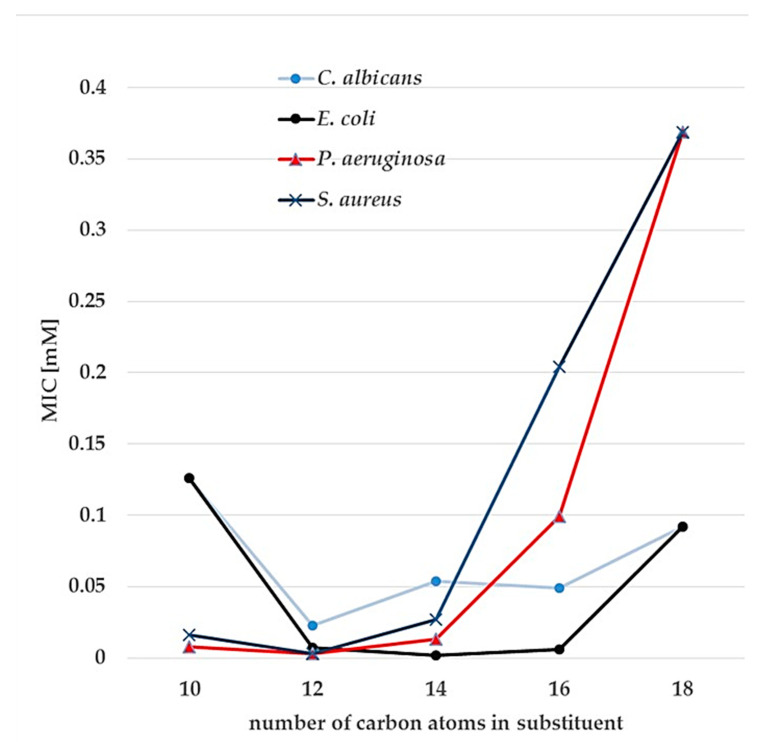
The relationships between MIC and the number of carbon atoms in the alkyl substituent for 3-methylaza-1,5-pentane-bis(*N*,*N*-dimethyl-*N*-alkylammonium) dibromides (**7**–**11**).

The highest MIC values against tested fungi were obtained for the derivatives with the shortest alkyl chains. The relationships between MIC and the number of carbon atoms in the alkyl substituent are of parabolic type (Figure 3), with the minimum around C12–C14; the highest antimicrobial activity was observed for the dodecyl and tetradecyl derivatives. It concerns also A. niger, which is usually more resistant to microbiocides than other microscopic fungi and it is very difficult to eradicate from any medical or food industry environment.

MIC values of 3-methylaza-1,5-pentane-bis(*N*,*N*-dimethyl-*N*-alkylammonium) dibromides against bacteria: *E. coli*, *P. aeruginosa*, and *S. aureus* were lower than those for microscopic fungi. Similarly, to the effects observed previously, the MIC values against bacteria at first decreased with the increasing length of the hydrocarbon chain, reached the lowest value for the dodecyl and tetradecyl substituent, and then increased again. The antimicrobial activity of dimeric alkylammonium salts is dependent on the length of the hydrocarbon chain. The shorter hydrocarbon chain is not able to penetrate the cell wall; an excessively long substituent is in turn too flexible and also cannot infiltrate the cell wall [30]. 

**Figure 4:**
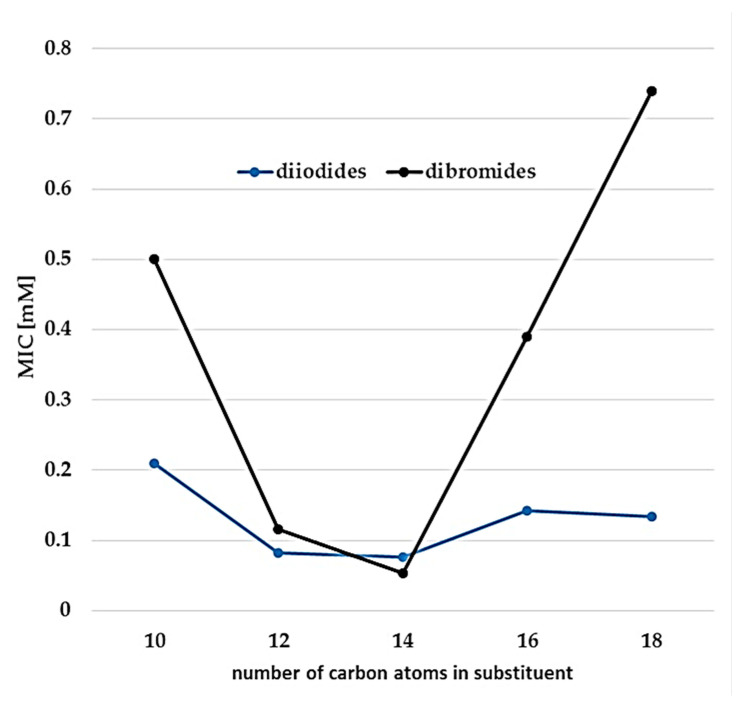
The relationship between MIC against A. niger and the number of carbon atoms in substituent for 3-methylaza-1,5-pentane-bis(*N*,*N*-dimethyl-*N*-alkylammonium) diiodides (**2**–**6**) and dibromides (**7**–**11**).

It is worth noting, that the antimicrobial activity of gemini surfactants depends not only on the length of the hydrocarbon chains or spacer but also on the type of counterion. In our research, it can be concluded that the diiodide MICs are generally lower than that of dibromides. However, around the smallest MIC for dodecyl and tetradecyl substituents, these values were very similar (Figure 4), which means that decontamination of any area can be done using optimized concentrations of 3-methylaza-1,5-pentane-bis(*N*,*N*-dimethyl-*N*-alkylammonium) dibromides and diiodides without the need to use a high concentration of microbiocides to effectively remove A. niger.

The MIC values against fungi and bacteria for 4-aza-1,7-heptane-bis(*N*,*N*-dimethyl-*N*-alkylammonium) diiodides (**19**–**21**) are placed in Table 3. Comparing MIC values against fungi of 3-methylaza-1,5-pentane-bis-(*N*,*N*-dimethyl-*N*-alkylammonium) diiodides (compounds **3**,**5**,**6**) and 4-aza-1,7-heptane-bis(*N*,*N*-dimethyl-*N*-alkylammonium) diiodides (compounds **19**–**21**) it was seen that gemini surfactants with a secondary amino group in the spacer have better antifungal activity. The lower MIC values against bacteria in this group of gemini surfactants show compounds with dodecyl substituents in the hydrophobic part. 

Gemini surfactants based on azapolymethylene spacers showed slightly lower MIC values in comparison to analogous gemini surfactants with polymethylene spacers. Classic gemini surfactant; hexamethylene-1,6-bis(*N*,*N*-dimethyl-*N*-dodecylammonium) dibromide shows MIC (mM): 0.12, 0.12, and 0.06 against *C. albicans*, *A. niger*, *P. chrysogenum*, and 0.087, 0.0073, and 0.0036 against *E. coli*, *P. aeruginosa* and *S. aureus*, respectively [36,37,38].

The MIC values against microorganisms of gemini surfactants with alkylaza-polymethylene spacers are shown in Table 4. Antifungal activity in this group of compounds grew with the increasing length of the middle chain, but differences between MIC values were not too large. The highest antibacterial activity was observed for compounds with the dodecyl substituent in the middle amine group for bromides. Antimicrobial activity of tested 4-alkylaza-1,7-heptane-bis(*N*,*N*-dimethyl-*N*-alkylammonium) diiodides is higher than for tested dibromide. This is probably due to the fact that dibromides have octadecyl chains at quaternary nitrogen atoms.

**Figure 5:**
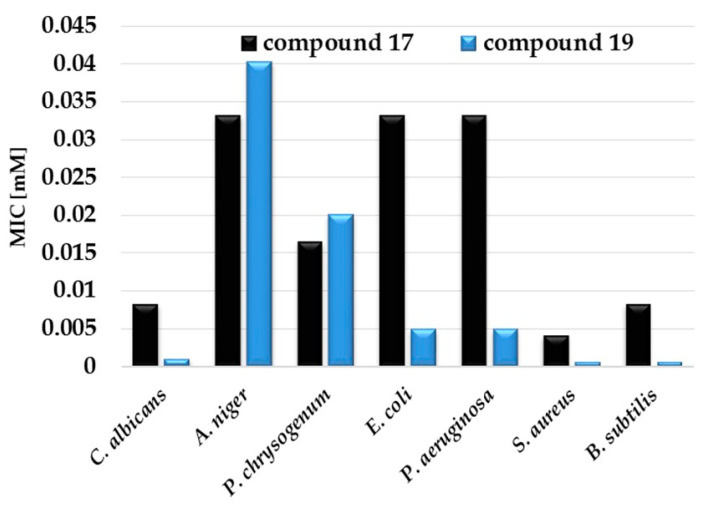
Comparison of two compounds, (**17**) and (**19**), having different hydrophobicity and their MIC (mM).

The comparison of 4-dodecylaza-1,7-heptane-bis(*N*,*N*-dimethyl-*N*-dodecylmmonium) diiodide (**17**) and 4-aza-1,7-heptane-bis(*N*,*N*-dimethyl-*N*-dodecylmmonium) diiodide (**19**) showed that lower MICs against all tested bacteria have got compounds with a secondary amine group in the spacer (Figure 5). On the other hand, the antifungal activity of **17** was lower than for **19**, except MIC against C. albicans. This is consistent with the fact that more hydrophobic compounds have a higher antifungal activity.

### 2.3. CMC Determination

The CMC was determined using a conductometric titration [39,40]. The ratio of the slopes above and below the CMC provided an estimation of the counterion binding parameter, β. The standard Gibbs energy of micellization (ΔG°_mic_) provides information that micelles formation is spontaneous and can be calculated using [14]:

(1) $${\Delta G{}^{\circ}}_{mic}=\;\mathrm{RT}\left(\frac{1}{2}+\beta \right)\mathrm{lncmc}\;–RT\ln2$$

where β is ionization degree, the CMC is expressed in mol/dm^3^, T is the temperature in Kelvin (K), and R is the gas constant.

Parameters describing micelles formation obtained from conductivity measurement for 3-methylaza-1,5-pentane-bis-(*N*,*N*-dimethyl-*N*-alkylammonium) salts and 4-aza-1,7-heptane-bis (*N*,*N*-dimethyl-*N*-alkylammonium) diiodides are presented in Table 5 and Table 6, respectively.

CMC of gemini surfactants depends on many factors such as alkyl chain length, number of quaternary nitrogen atoms, counterion, functionalization of the spacer, and hydrophobic chain [15,30,40,41]. CMC decreases as the length of the hydrocarbon chain increases. For example, an increase in the length of the hydrocarbon chain from 8 to 18 carbon atoms causes a decrease in CMC of 3.46 to 0.06 mM (compounds (**1**–**6**)). This is in agreement with data presented previously with regard to the relationship between gemini surfactants with the polymethylene linker and spacer functionalized with an ether group [42]. Comparing the effect of the counterion, it can be stated that bromides have lower CMC than analogous iodides. 

The values of the counterion binding parameter (β) provide information about an average number of counterions per surfactant ion in the micelle [43,44]. For compounds in the same homologous series, β increases with the elongation of the alkyl chain, reaching the highest value for dodecyl derivative, and then decreases with the elongation of the alkyl chain. This correlation is characteristic of gemini surfactants [42]. The big value of β is caused by the stronger binding of the counterion to the micelles, suggesting closer packing of the hydrophilic parts of surfactant and higher surface charge density at the micelle solution interface [42]. The standard Gibbs energy values of the micellization process (ΔG°mic) for gemini surfactants were negative. This means that the micellization process was spontaneous. In addition, ΔG°mic decreases with the elongation of the alkyl chain indicating that the aggregation process is driven by the hydrophobic part of surfactants. 

In a group of 4-aza-1,7-heptane-bis(*N*,*N*-dimethyl-*N*-alkylammonium) diiodides dependencies CMC was similar to those described earlier; CMC decreased as the length of the hydrocarbon chain increased.

In the case of 4-alkylaza-1,7-heptane-bis(*N*,*N*-dimethyl-*N*-alkyl-ammonium) salts, another long hydrophobic chain was introduced to the molecule, significantly affecting CMC of gemini surfactants (Table 7). 

**Figure 6:**
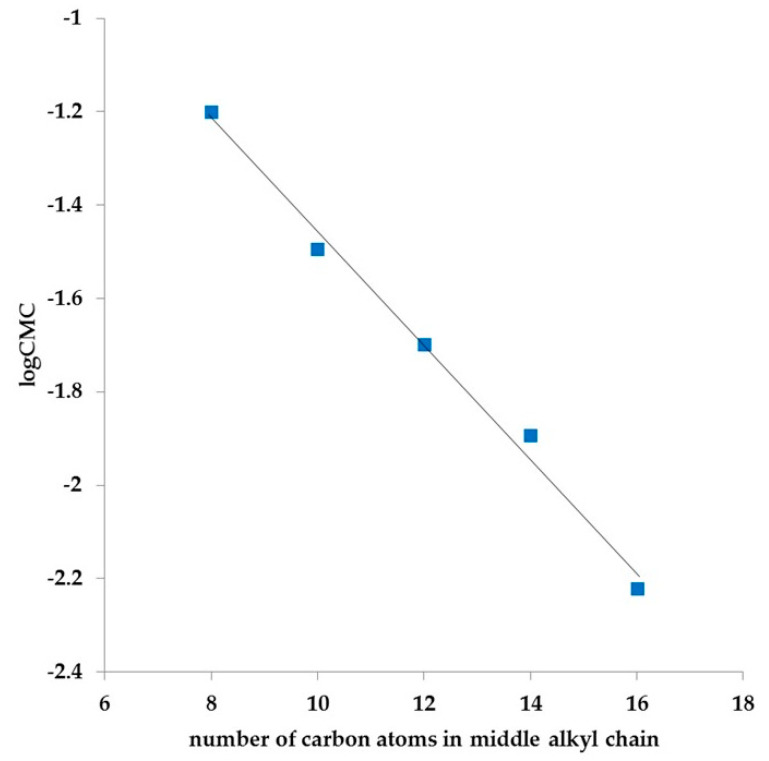
Variation in log CMC with the number of carbon atoms in the middle hydrocarbon chain for 4-alkylaza-1,7-heptane-bis (*N*,*N*-dimethyl-*N*-alkylammonium) dibromides (**12**–**16**).

Based on the data presented, it can be noted that the introduction of additional long hydrocarbon chains to the molecule, causes increases in surface activity and decreases in CMC values. The relationship between log CMC and the number of carbon atoms in a middle hydrophilic chain decreased linearly (Figure 6), analogous to conventional gemini surfactants [42].

The counterion binding parameter for gemini surfactants with additional alkyl chains decreases with the elongation of the central alkyl chain. When β is the smallest, the counterion is more strongly bound by aggregates, creating more compacted micelles because of the increasing shielding of the electrostatic charges of the counterion and the cationic part of the surfactant. Gibbs free energy of micellization of this group of gemini surfactants was negative, which clearly shows that the micellization process is spontaneous. The longer the middle alkyl chain, the greater the tendency to form micelles.

There was no simple correlation between MIC and CMC values [45], but for compounds with short or medium chains (**1**–**4**, **7**–**9**, **19**), the MICs against all tested microorganisms were lower than CMC. In other cases, for gemini with hexadecyl or octadecyl hydrocarbon chains (**5**, **6**, **10**, **11**–**18**, **20**, **21**) MICs were comparable or higher than CMC, due to their lower biocidal activity than gemini with the dodecyl substituent.

## 3. Materials and Methods 

### 3.1. Materials Used

*N*,*N*,*N*′,*N*′,*N*″-pentamethyl-1,4,7-triazaheptane, 3,3′-iminobis(*N*,*N*-dimethylpropylamine), all *n*-akylbromides and *n*-alkyliodides were purchased from Sigma-Aldrich (Poznań, Poland). Purity of reagents was at least 95%. Acetonitrile was purchased from VWR chemicals; purity for HPLC, and potassium carbonate was bought from P.P.H. Stanlab (Lublin, Poland). All reagents and solvents were obtained without further purification.

### 3.2. Experimental Methods 

#### 3.2.1. Spectroscopic and Spectrometry Characterization of Gemini Surfactants

The NMR spectra were measured with a Varian model VNMR-S 400 MHz (Oxford, UK) operating at 403 and 101 MHz for ^1^H and ^13^C, respectively. The ^13^C and ^1^H chemical shifts were measured in CDCl_3_ and TFA-d relative to an internal standard of TMS. All proton and carbon-13 resonances were assigned by ^1^H(COSY) and ^13^C (HETCOR). All 2D NMR spectra were recorded at 298 K on a Bruker Avance DRX 600 spectrometer (Billerica, MA, USA) operating at the frequencies 600.315 MHz (1H) and 150.963 MHz (13C) and equipped with a 5 mm triple-resonance inverse probe head (1H/31P/BB) with a self-shielded *z* gradient coil (90° 1H pulse width 9.0 μs and 13 C pulse width 13.3 μs).

The ESI (electron spray ionization) mass spectra and elemental analysis were recorded on a Waters/Micromass (Manchester, UK) ZQ mass spectrometer equipped with a Harvard Apparatus (Saint Laurent, Canada) syringe pump. The sample solutions were prepared in methanol at a concentration of approximately 10^−5^ M. The standard ESI-MS mass spectra were recorded at the cone voltage 90 V.

#### 3.2.2. Elemental Analysis 

The measurements were carried out on a FLASH 2000 elemental analyzer (Delft, Netherlands) with a thermal conductivity detector.

#### 3.2.3. Conductivity Measurement

Conductivity measurements were carried out using a (CO 300, VWR, Gdańsk, Poland) Conductivity Meter with two-pole measuring cell (Ø 12 mm, stainless steel). The instrument was calibrated using a standard (147 µS/cm at 25 °C). Deionized double distilled water was used in all the experimental work, and its specific conductivity value was around 1–2 × 10^−6^ S/cm. 

#### 3.2.4. MIC Determination

Gemini compounds were tested for antimicrobial activity against bacteria: *Escherichia coli* ATCC 10536, *Pseudomonas aeruginosa* ATCC 85327, *Staphylococcus aureus* ATCC 6538, *Bacillus subtilis* ATCC 6633, and fungi: *Candida albicans* ATCC 10231, *Aspergillus niger* ATCC 16401, and *Penicillium chrysogenum* ATCC 60739. The MIC values for bacteria and yeast were determined by a tube standard two-fold dilution method [36]. The 24-h culture of bacteria in TSB medium and yeast culture in MEB medium were centrifuged (8000 rpm, for 10 min), resuspended in physiological salt solution, and diluted 100-fold (10^7^ cfu/mL bacteria and 10^6^ cfu/mL yeast). One milliliter of microorganism suspension was mixed with 1 mL of media (TSB for bacteria, MEB for yeast) containing serial dilutions of the tested compounds and incubated at 37 °C for 24 h—bacteria, 48 h—yeast. As a growth control, a suspension of microorganisms in TSB/MEB medium without the biocides was used. The MICs were defined as the lowest concentration of the compounds at which there was no visible growth. The test was repeated three times.

## 4. Conclusions

Derivatives of 3-methylaza-1,5-pentane-bis(*N*,*N*-dimethyl-*N*-alkylammonium) salts, 4-aza-1,7-heptane-bis(*N*,*N*-dimethyl-*N*-alkylammonium) diiodides, and 4-alkyl-aza-1,7-heptane-bis(*N*,*N*-dimethyl-*N*-alkylammonium) salts were obtained in the reaction of alkylamines with *n*–bromoalkanes or *n*–iodoalkanes, respectively. The structures of the title compounds were confirmed by ^1^H and ^13^C NMR, ESI-MS spectra and elemental analysis, and HMBC and HSQC spectra. We proved that the MIC of gemini surfactants with azapolymethylene spacers reached the minimum value for tetradecyl and dodecyl derivatives. CMC of the synthesized surfactants are very low and strongly depends on the length of the carbon chain; the longer the hydrocarbon chain, the lower the CMC values. Gemini surfactants with additional hydrocarbon chains in the spacer show a lower CMC value, and lower MIC against fungi, while higher MIC values against bacteria in comparison to the derivative with the amine group in the spacer were observed.

## Figures and Tables

**Table 1:** Minimal inhibitory concentrations against fungi of 3-methylaza-1,5-pentane-bis-(*N*,*N*-dimethyl-*N*-alkylammonium) diiodides.

Compound	Minimal Inhibitory Concentration (mM)		
	*C. albicans*	*A. niger*	*P. chrysogenum*
**1**	0.960	1.914	0.475
**2**	0.110	0.210	0.110
**3**	0.041	0.082	0.041
**4**	0.037	0.076	0.076
**5**	0.071	0.142	0.071
**6**	0.268	0.134	0.134

**Table 2:** Minimal inhibitory concentrations against fungi and bacteria of 3-methylaza-1,5-pentane-bis-(*N*,*N*-dimethyl-*N*-alkylammonium) dibromides.

Compound	Minimal Inhibitory Concentration (mM)				
	*C. albicans*	*A. niger*	*E. coli*	*P. aeruginosa*	*S. aureus*
**7**	0.126	0.500	0.126	0.008	0.016
**8**	0.023	0.116	0.007	0.003	0.003
**9**	0.054	0.054	0.002	0.013	0.027
**10**	0.049	0.390	0.006	0.099	0.204
**11**	0.092	0.739	0.092	0.369	0.369

**Table 3:** Minimal inhibitory concentrations against fungi and bacteria of 4-aza-1,7-heptane-bis(*N*,*N*-dimethyl-*N*-alkylammonium) diiodides.

Compound		Minimal Inhibitory Concentration (mM)					
	*C. albicans*	*A. niger*	*P. chrysogenum*	*E. coli*	*P. aeruginosa*	*S. aureus*	*B. subtilis*
**19**	0.010	0.0403	0.0201	0.0050	0.0050	0.0006	0.0006
**20**	0.0353	0.0701	0.0701	0.0701	0.0701	0.0087	0.0085
**21**	0.0165	0.0334	0.0165	0.0342	0.0659	0.0165	0.0165

**Table 4:** Minimal inhibitory concentrations against fungi and bacteria of 4-alkylaza-1,7-heptane-bis(*N*,*N*-dimethyl-*N*-alkylammonium) dibromides (**12**–**16**) and diiodides (**17–18**).

Compound		Minimal Inhibitory Concentration (mM)					
	*C. albicans*	*A. niger*	*P. chrysogenum*	*E. coli*	*P. aeruginosa*	*S. aureus*	*B. subtilis*
**12**	0.0647	0.1787	0.0647	0.0647	0.0647	0.0162	0.0647
**13**	0.0317	0.1257	0.0628	0.0317	0.0628	0.0157	0.0628
**14**	0.0308	0.1222	0.0611	0.0153	0.0308	0.0076	0.0308
**15**	0.0299	0.1189	0.0594	0.0299	0.0299	0.0299	0.0594
**16**	0.0292	0.0579	0.0292	0.0292	0.0292	0.0292	0.0292
**17**	0.0082	0.0332	0.0165	0.0332	0.0332	0.0041	0.0082
**18**	0.0141	0.0559	0.0282	0.0559	0.0559	0.0282	0.0282

**Table 5:** Critical micelle concentration (CMC), counterion binding parameter (β) and Gibbs free energy of micellization (ΔG°_mic_) of 3-methylaza-1,5-pentane-bis-(*N*,*N*-dimethyl-*N*-alkyl-ammonium) diiodides (**1**–**6**) and dibromides (**7**–**11**) at 25 °C.

Compound Number	CMC (mM)	β	ΔG°_mic_ (kJ/mol)
**1**	3.46 ± 0.11	0.44	−14.91
**2**	2.46 ± 0.12	0.45	−21.81
**3**	0.75 ± 0.06	0.52	−18.12
**4**	0.35 ± 0.02	0.51	−19.66
**5**	0.11 ± 0.01	0.43	−22.72
**6**	0.06 ± 0.01	0.43	−24.10
**7**	2.78 ± 0.13	0.38	−14.55
**8**	1.06 ± 0.09	0.53	−38.20
**9**	0.48 ± 0.07	0.51	−20.27
**10**	0.15 ± 0.02	0.37	−24.28
**11**	0.08 ± 0.01	0.29	−40.20

**Table 6:** Critical micelle concentration (CMC), counterion binding parameter (β), and Gibbs free energy of micellization (ΔG°_mic_) of 4-aza-1,7-heptane-bis(*N*,*N*-dimethyl-*N*-alkylammonium) diiodides at 25 °C.

Compound Number	CMC (mM)	Β	ΔG°_mic_ (kJ/mol)
**19**	1.001 ± 0.121	0.84	−22.53
**20**	0.321 ± 0.050	0.49	−22.30
**21**	0.021 ± 0.003	0.81	−39.05

**Table 7:** Critical micelle concentration (CMC), counterion binding parameter (β), and Gibbs free energy of micellization (ΔG°_mic_) of 4-alkylaza-1,7-heptane-bis(*N*,*N*-dimethyl-*N*-alkylammonium) dibromides (**12**–**16**) and diiodides (**17,18**) at 25 °C.

Compound Number	CMC (mM)	β	ΔG°_mic_ (kJ/mol)
**12**	0.063 ± 0.003	0.65	−30.80
**13**	0.032 ± 0.002	0.40	−26.19
**14**	0.020 ± 0.001	0.37	−26.30
**15**	0.013 ± 0.002	0.37	−27.31
**16**	0.006 ± 0.001	0.31	−26.54
**17**	0.064 ± 0.003	0.43	−25.17
**18**	0.033 ± 0.004	0.42	−26.51

## Supplementary Materials

The following are available online at https://www.mdpi.com/1420-3049/25/18/4054/s1, Supplementary Materials contain details on the synthesis and analysis of compounds.
