# Supplementary material for: Antimicrobial Activity of Gemini Surfactants with Azapolymethylene Spacer
Source: Molecules. 2020 Sep 4;25(18):4054. doi: 10.3390/molecules25184054 (PMC7571221; doi:10.3390/molecules25184054)

SUPPLEMENTARY MATERIAL

Preparation of compound (1)

To a solution of *N,N,N′,N′,N″*-pentamethyl-1,4,7-triazaheptane (0.01 mol; 2g) in acetonitrile (50 mL) was addend *n*-iodooctane (0.02 mol; 5.52g). The reaction mixture was heated under reflux for 32hours. The solvent was evaporated under reduced pressure and the residue was dried over P_4O_{10} and then crystallized from acetone. White solid, mp: 156–158 °C, yield 43%.

^{1}H NMR (403 MHz, $CDCl_3$): δ 3.98-3.84 (m, 4H), 3.71-3.56 (m, 4H), 3.43 (s, 12H), 3.25-3.11 (m, 4H), 2.55 (s, 3H), 1.73-1.67 (m, 4H), 1.48-1.19 (m, 24H), 0.88 (t, 6H).**^{13}C NMR (101 MHz, $CDCl_3$)** δ 65.45, 61.18, 51.51, 50.93, 43.05, 31.49, 29.05, 28.90, 26.04, 22.81, 22.42, 22.19, 13.94. **ESI-MS** (*m*/*z*) 200 $[C_{25}H_{57}N_3]^{2+}$

Preparation of compound (2)

To a solution of *N,N,N′,N′,N″*-pentamethyl-1,4,7-triazaheptane (0.02 mol; 3.75 g) in acetonitrile (50 mL) was addend *n*-iododecane (0,05 mol; 13 g). The reaction mixture was heated under reflux for 23hours. The solvent was evaporated under reduced pressure and the residue was dried over P_4O_{10} and then crystallized from acetone. White solid, mp: 202–204 °C, yield 48%.

^{1}HNMR (403 MHz, $CDCl_3$): δ 3.98-3.85 (m, 4H), 3.70-3.56 (m, 4H), 3.43 (s, 12H), 3.25-3.12 (m, 4H), 2.55 (s, 3H), 1.84-1.67 (m, 4H), 1.48-1.18 (m, 32 H), 0.88 (t, 6H).**^{13}C NMR (101 MHz, $CDCl_3$)** 65.52, 61.25, 51.54, 50.95, 43.11, 31.71, 29.33, 29.30, 29.18, 29.13, 26.10, 22.86, 22.52, 14.00. **Elemental analysis found (calc)** %C 48.58 (49.08); %H 9.68 (9.23); %N 5.48 (5.92)

Preparation of compound (3)

To a solution of *N,N,N′,N′,N″*-pentamethyl-1,4,7-triazaheptane (0.02 mol; 3.75 g) in acetonitrile (50 mL) was addend *n*-iodododecane (0,05 mol; 13 g). The reaction mixture was heated under reflux for 23hours. The solvent was evaporated under reduced pressure and the residue was dried over P_4O_{10} and then crystallized from acetone. White solid, mp: 211–212 °C, yield 46%.

^{1}H NMR (403 MHz, $CDCl_3$): δ 3.99-3.86 (m, 4H), 3.68-3.55 (m, 4H), 3.43 (s, 12H), 3.24-3.12 (m, 4H), 2.55 (s, 3H), 1.83-1.68 (m, 4H), 1.48-1.18 (m, 40 H), 0.88 (t, 6H). **^{13}C NMR (101 MHz, $CDCl_3$)** 65.54, 61.27, 51.54, 50.95, 43.12, 26.12, 22.87, 14.01 **ESI-MS** (m/z) 255 $[C_{33}H_{73}N_3]^{2+}$

Preparation of compound (4)

To a solution of *N,N,N′,N′,N″*-pentamethyl-1,4,7-triazaheptane (0.01 mol; 2 g) in acetonitrile (50 mL) was addend *n*-iodotetradecane (0.03 mol; 9.22 g). The reaction mixture was heated under reflux for 4hours. The solvent was evaporated under reduced pressure and the residue was dried over P_4O_{10} and then crystallized from acetone. White solid, mp: 209-210 °C, yield 85%.

^{1}H NMR (403 MHz, $CDCl_3$): δ 3.99-3.86 (m, 4H), 3.68-3.54 (m, 4H), 3.42 (s, 12H), 3.25-3.13 (m, 4H), 2.55 (s, 3H), 1.85-1.66 (m, 4H), 1.49-1.17 (m, 48 H), 0.88 (t, 6H). **^{13}C NMR (101 MHz, $CDCl_3$)**:65.57, 61.31, 51.56, 50.97, 43.13, 26.14, 22.89, 14.00 **Elemental analysis found (calc)** %C 53.43 (54.01); %H 10.38 (10.04); %N 4.78 (5.11)

<u>Preparation of compound (5)</u>

To a solution of *N,N,N′,N′,N″*-pentamethyl-1,4,7-triazaheptane (0.01 mol; 2 g) in acetonitrile (50 mL) was addend *n*-iodohexadecane (0.02 mol; 7.5 g). The reaction mixture was heated under reflux for 4hours. The solvent was evaporated under reduced pressure and the residue was dried over P_4O_{10} and then crystallized from acetonitrile. White solid, mp: 215-217 °C, yield 42%.

^{1}H NMR (403 MHz, $CDCl_3$): δ 3.98-3.86 (m, 4H), 3.68-3.55 (m, 4H), 3.42 (s, 12H), 3.24-3.13 (m, 4H), 2.56 (s, 3H), 1.83-1.68 (m, 4H), 1.49-1.17 (m, 56 H), 0.88 (t, 6H). **^{13}C NMR (101 MHz, $CDCl_3$)**:65.57, 61.31, 51.55, 50.97, 43.15, 26.14, 22.89, 14.02. **Elemental analysis found (calc)** %C 55.37 (56.09); %H 10.12 (10.18); %N 4.45 (4.79)

<u>Preparation of compound (6)</u>

To a solution of *N,N,N′,N′,N″*-pentamethyl-1,4,7-triazaheptane (0.01 mol; 2 g) in acetonitrile (50 mL) was addend *n*-iodooctadecane (0.02 mol; 8 g). The reaction mixture was heated under reflux for 2hours. The solvent was evaporated under reduced pressure and the residue was dried over P_4O_{10} and then crystallized from acetone:acetonitrile (1:1). White solid, mp: 209–211 °C, yield 65%

^{1}HNMR (403 MHz, $CDCl_3$): δ 3.98-3.87 (m, 4H), 3.68-3.56 (m, 4H), 3.42 (s, 12H), 3.26-3.13 (m, 4H), 2.56 (s, 3H), 1.83-1.67 (m, 4H), 1.49-1.17 (m, 64 H), 0.88 (t, 6H). **^{13}C NMR (101 MHz, $CDCl_3$)** δ 65.58, 61.32, 51.56, 50.97, 43.15, 31.81, 29.62, 29.56, 29.46, 29.37, 29.25, 26.15, 22.89, 22.58, 14.02. **Elemental analysis found (calc)** %C 58.09 (57.86); %H 10.99 (10.47); %N 4.24 (4.50)

<u>Preparation of compound (7)</u>

To a solution of *N,N,N′,N′,N″*-pentamethyl-1,4,7-triazaheptane (0.02 mol; 3.75 g) in acetonitrile (50 mL) was addend *n*-bromodecane (0.04 mol; 8.3 mL). The reaction mixture was heated under reflux for 60hours. The solvent was evaporated under reduced pressure and the residue was dried over P_4O_{10} and then crystallized from acetonitrile. White solid, mp 218-220 °C, yield 48%.

^{1}H NMR (403 MHz, $CDCl_3$): δ 4.00-3.91 (m, 4H), 3.65-3.55 (m, 4H), 3.43 (s, 12H), 3.21-3.13 (m, 4H), 2.53 (s, 3H), 1.79-1.66 (m, 4H), 1.44-1.18 (m, 32 H), 0.88 (t, 6H).

^{13}C NMR (101 MHz, $CDCl_3$) δ 65.39, 61.92, 61.22, 51.21, 50.77, 48.52, 45.13, 43.09, 31.76, 29.36, 29.33, 29.23, 29.17, 26.23, 22.86, 22.58, 14.04. **Elemental analysis found (calc)** %C 56.61 (56.58); %H 10.62 (10.64); %N 6.82 (6.83)

Preparation of compound (8)

To a solution of *N,N,N′,N′,N″*-pentamethyl-1,4,7-triazaheptane (0.02 mol; 3.75 g) in acetonitrile (50 mL) was addend *n*-bromododecane (0.04 mol; 9.3 mL). The reaction mixture was heated under reflux for 12hours. The solvent was evaporated under reduced pressure and the residue was dried over P_4O_{10} and then crystallized from acetonitrile. White solid, mp 225-227 °C, yield 42%.

^{1}H NMR (403 MHz, TFA-d): δ 4.00-3.90 (m, 4H), 3.65-3.53 (m, 4H), 3.41 (s, 12H), 3.23-3.11 (m, 4H), 2.53 (s, 3H), 1.80-1.65 (m, 4H), 1.45-1.19 (m, 40 H), 0.88 (t, 6H). **^{13}C NMR (101 MHz, TFA-d)** δ 66.08, 62.06, 53.62, 52.30, 49.48, 43.57, 34.02, 32.04, 31.94, 31.79, 31.61, 31.32, 28.23, 24.66, 15.82. **Elemental analysis found (calc)** %C 58.22 (58.22); %H 11.12 (10.96); %N 6.21 (6.17)

Preparation of compound (9)

To a solution of *N,N,N′,N′,N″*-pentamethyl-1,4,7-triazaheptane (0.02 mol; 3.75 g) in acetonitrile (50 mL) was addend *n*-bromotetradecane (0.04 mol; 12 mL). The reaction mixture was heated under reflux for 10hours. The solvent was evaporated under reduced pressure and the residue was dried over P_4O_{10} and then crystallized from acetonitrile. White solid, mp 229-231 °C, yield 55%.

^{1}H NMR (403 MHz, $CDCl_3$): δ 4.03-3.92 (m, 4H), 3.68-3.54 (m, 4H), 3.42 (s, 12H), 3.23-3.12 (m, 4H), 2.54 (s, 3H), 1.81-1.65 (m, 4H), 1.46-1.19 (m, 48 H), 0.88 (t, 6H).

^{13}C NMR (101MHz, $CDCl_3$) δ 65.35, 61.54, 61.13, 51.15, 50.74, 48.29, 44.79, 43.05, 31.79, 29.56, 29.53, 29.49, 29.39, 29.31, 29.23, 26.20, 22.83, 22.56, 14.00. **Elemental analysis found (calc)** %C 59.65 (59.58); %H 11.14 (11.22); %N 5.71 (5.63)

Preparation of compound (10)

To a solution of *N,N,N′,N′,N″*-pentamethyl-1,4,7-triazaheptane (0.02 mol; 3.75 g) in acetonitrile (50 mL) was addend *n*-bromoheksadecane (0.04 mol; 12.4 mL). The reaction mixture was heated under reflux for 8hours. The solvent was evaporated under reduced pressure and the residue was dried over P_4O_{10} and then crystallized from acetonitrile. White solid, mp 223-225 °C, yield 49%.

^{1}H NMR (403 MHz, $CDCl_3$): δ 4.02-3.92 (m, 4H), 3.68-3.56 (m, 4H), 3.42 (s, 12H), 3.24-3.11 (m, 4H), 2.54 (s, 3H), 1.81-1.64 (m, 4H), 1.46-1.19 (m, 56 H), 0.88 (t, 6H). **^{13}C NMR (101 MHz, $CDCl_3$)** δ 65.39, 61.93, 61.23, 51.16, 50.81, 48.51, 45.12, 43.09, 31.82, 29.60, 29.56, 29.53, 29.42, 29.34, 29.26, 29.13, 26.22, 22.85, 22.59, 14.03. **Elemental analysis found (calc)** %C 62.28 (62.10); %H 10.71 (11.44); %N 5.30 (5.30)

Preparation of compound (11)

To a solution of *N,N,N′,N′,N″*-pentamethyl-1,4,7-triazaheptane (0.02 mol; 3.75 g) in acetonitrile (50 mL) was addend *n*-bromooctadecane (0.04 mol; 13.4 mL). The reaction mixture was heated under reflux for 2hours. The solvent was evaporated under reduced pressure and the residue was dried over P_4O_{10} and then crystallized from acetone: acetonitrile (1:2). White solid, mp 256-259 °C, yield 59%.

^{1}H NMR (403 MHz, $CDCl_3$): δ 4.02-3.92 (m, 4H), 3.67-3.55 (m, 4H), 3.42 (s, 12H), 3.22-3.12 (m, 4H), 2.53 (s, 3H), 1.79-1.65 (m, 4H), 1.45-1.19 (m, 64 H), 0.88 (t, 6H).

^{13}C NMR (101 MHz, $CDCl_3$) δ 65.39, 61.22, 51.17, 50.79, 48.51, 43.10, 31.82, 29.61, 29.58, 29.57, 29.54, 29.43, 29.34, 29.26, 29.25, 26.23, 22.86, 22.59, 14.03. **Elemental analysis found (calc)** %C 63.71 (63.65); %H 11.83 (11.63); %N 4.91 (4.95)

Preparation of compound (12)

To a solution of 4-aza-1,7-heptane-bis(*N,N*-dimethyl-*N*-otadecylammonium) dibromide(0.005 mol; 4.27 g) in acetonitrile (20 mL) was added *n*-bromooctane (0.006 mol; 1.16 g) and sodium carbonate (0.006 mol; 0.64 g). The reaction mixture was heated under reflux for 28hours. The solvent was evaporated under reduced pressure and the residue was dried over P_4O_{10} and then crystallized from acetonitrile.White solid, mp 67-68 °C, yield 55%.

^{1}H NMR (403 MHz, $CDCl_3$): δ 3.93 (m, 4H), 3.38 (m, 12H), 2.72 (t, 4H), 2.52 (m, 4H), 3.43 (m, 6H), 1.74 (m, 6H), 1.35 (m, 12H), 1.26 (m, 54H), 0.87 (t, 9H).

^{13}C NMR (101 MHz, $CDCl_3$) δ 65.64, 64.73, 62.72, 61.87, 54.17, 51.08, 50.81, 49.73, 44.51, 31.87, 31.77, 31.60, 30.43, 29.67, 29.6, 29.59, , 29.48, 29.41, 29.32, , 29.10, 27.36, 26.30, 25.38, 22.84, 22.64, 20.14, 14.08. **ESI-MS** (*m/z*) 403 $[C_{54}H_{115}N_3]^{2+}$, 347 $[C_{46}H_{98}N_3]^{2+}$

Preparation of compound (13)

To a solution of 4-aza-1,7-heptane-bis(*N,N*-dimethyl-*N*-otadecylammonium) dibromide (0.005 mol; 4.27 g) in acetonitrile (20 mL) was added *n*-bromodecane (0.006 mol; 1.33 g) and sodium carbonate (0.006 mol; 0.64 g). The reaction mixture was heated under reflux for 24hours. The solvent was evaporated under reduced pressure and the residue was dried over P_4O_{10} and then crystallized from acetonitrile. White solid, mp 65-66 °C, yield 60%.

^{1}H NMR (403 MHz, $CDCl_3$): δ 3.92 (m, 4H), 3.38 (m, 12H), 2.71 (t, 4H), 2.30(m, 4H), 3.42(m, 6H), 1.74 (m, 6H), 1.35 (m, 12H), 1.26 (m, 58H), 0.88 (t, 9H).**^{13}C NMR (101 MHz, $CDCl_3$)** δ 64.78, 62.60, 54.23, 51.13, 50.82, 49.70, 31.87, 29.67, 29.48, 29.14, 29.31, 29.24, 27.32, 26.30, 25.36, 22.84, 22.64, 20.05, 14.07. **ESI-MS** (*m/z*) 417 $[C_{56}H_{119}N_3]^{2+}$, 347 $[C_{46}H_{98}N_3]^{2+}$

Preparation of compound (14)

To a solution of 4-aza-1,7-heptane-bis(*N,N*-dimethyl-*N*-otadecylammonium) dibromide (0.005 mol; 4.27 g) in acetonitrile (20 mL) was added *n*-bromododecane (0.006 mol; 1.49 g) and sodium carbonate (0.006 mol; 0.64 g). The reaction mixture was heated under reflux for 22hours. The solvent was evaporated under reduced pressure and the residue was dried over P_4O_{10} and then crystallized from acetonitrile. White solid, mp 62-63 °C, yield 72 %.

^{1}H NMR (403 MHz, $CDCl_3$): δ 3.93 (m, 4H), 3.39 (s, 12H), 2.77 (t, 4H), 2.22 (m, 4H), 3.45 (m, 6H), 1.74 (m, 6H), 1.35 (m, 12H), 1.26 (m, 62H), 0.88 (t, 9H).**^{13}C NMR (101 MHz, $CDCl_3$)** δ 64.85, 62.48, 54.28, 51.09, 50.79, 49.54, 31.87, 29.67, 29.61, 29.48, 29.41, 29.31, 29.24, 27.27, 26.28, 25.30, 22.83, 22.63, 19.89, 14.08.

Preparation of compound (15)

To a solution of 4-aza-1,7-heptane-bis(*N,N*-dimethyl-*N*-otadecylammonium) dibromide (0.005 mol; 4.27 g) in acetonitrile (20 mL) was added *n*-bromotetradecane (0.005 mol; 1.67 g) and sodium carbonate (0.006 mol; 0.64 g). The reaction mixture was heated under reflux for 20hours. The solvent was evaporated under reduced pressure and the residue was dried over P_4O_{10} and then crystallized from acetonitrile. White solid, mp 60-61 °C, yield 75%.

^{1}H NMR (403 MHz, $CDCl_3$): δ 3.91 (m, 4H), 3.42 (s, 12H), 2.81 (t, 4H), 2.07 (m, 4H), 3.47 (m, 6H), 1.74 (m, 6H), 1.34 (m, 12H), 1.26 (m, 64H), 0.88 (t, 9H)**^{13}C NMR (101 MHz, $CDCl_3$)** δ 64.47, 63.00, 53.96, 51.06, 50.03, 31.87, 29.67, 29.61, 29.48, 29.40, 29.31, 29.24, 27.48, 26.30, 25.56, 22.84, 22.64, 20.54, 14.08. **ESI-MS** (*m*/*z*) 445 $[C_{60}H_{127}N_3]^{2+}$, 347 $[C_{46}H_{98}N_3]^{2+}$

Preparation of compound (16)

To a solution of 4-aza-1,7-heptane-bis(*N,N*-dimethyl-*N*-otadecylammonium) dibromide (0.006 mol; 5.12 g) in acetonitrile (20 mL) was added *n*-bromoheksadecane (0.005 mol; 2.19 g) and sodium carbonate (0.006 mol; 0.64 g). The reaction mixture was heated under reflux for 16hours. The solvent was evaporated under reduced pressure and the residue was dried over P_4O_{10} and then crystallized from acetonitrile. White solid, mp 57–59 °C, yield 78%.

^{1}H NMR (403 MHz, $CDCl_3$): 3.91 (m, 4H), 3.41 (s, 12H), 2.78 (t, 4H), 2.04 (m, 4H), 3.41 (m, 6H), 1.73 (m, 6H), 1.34 (m, 12H), 1.25 (m, 66H), 0.88 (t, 9H) δ **^{13}C NMR (101 MHz, $CDCl_3$)** δ 64.39, 63.07, 53.92, 51.03, 50.08, 31.87, 29.66, 29.61, 29.46, 29.39, 29.31, 29.24, 27.49, 26.30, 25.58, 22.83, 22.63, 20.60, 14.07. **ESI-MS** (*m*/*z*) 459 $[C_{62}H_{135}N_3]^{2+}$, 347 $[C_{46}H_{98}N_3]^{2+}$

Preparation of compound (17)

To a solution of 4-aza-1,7-heptane-bis(*N*,*N*-dimethyl-*N*-dodecylammonium) diiodide(0.005 mol; 4 g) in acetonitrile (20 mL) was added *n*-iodododecane (0.015 mol; 4.45 g) and sodium carbonate (0.006 mol; 0.64 g). The reaction mixture was heated under reflux for 20hours. The solvent was evaporated under reduced pressure and the residue was dried over P_4O_{10} and then crystallized from acetonitrile. White solid, mp 110-111 °C, yield 60%.

^{1}H NMR (403 MHz, $CDCl_3$): δ 4.031-3.902 (m, 4H), 3.57-3.47 (m, 4H), 3.87 (s, 12H), 2.67-2.64 (t, 4H), 2.47-2.44 (t, 2H), 1.40 (s, 6H), 1.26 (s, 50 H), 0.87 (t, 9H). **^{13}C NMR (101 MHz, $CDCl_3$)** δ 64.38, 63.55, 53.29, 51.12, 50.21, 31.76, 29.48, 27,60, 26.17, 25.49, 22.86, 22.55, 21.12, 13.99.

Preparation of compound (18)

To a solution of 4-aza-1,7-heptane-bis(*N*,*N*-dimethyl-*N*-hexadecylammonium) dibromide (0.005 mol; 4 g) in acetonitrile (20 mL) was added *n*-iodohexadecane (0.015 mol; 5.30 g) and sodium carbonate (0.006 mol; 0.64 g). The reaction mixture was heated under reflux for 22hours. The solvent was evaporated under reduced pressure and the residue was dried over P_4O_{10} and then crystallized from acetonitrile. White solid, mp 65-67 °C, yield 65%.

^{1}H NMR (403 MHz, $CDCl_3$): δ 4.04-3.98 (m, 4H), 3.50-3.45 (m, 4H), 3.38 (s, 12H), 2.67 (s, 4H), 2.47-2.44 (t, 2H), 2.02 (s, 4H), 1.75 (s, 6H), 1.36 (s, 6 H), 1.25 (s, 36H), 0.88 (s, 9H) **^{13}C NMR (101 MHz, $CDCl_3$)** δ 64.42, 63.68, 53.21, 51.09, 50.31, 31.85, 29.63, 29.59, 29.55, 27.65, 26.22, 25.38, 22.89, 22.61, 21.17. **ESI-MS** (*m*/*z*) 347 $[C_{46}H_{99}N_3]^{2+}$, 440 $[C_{28}H_{62}N_3]^+$

Preparation of compound (19)

To a solution of 3,3′-Iminobis(*N*,*N*-dimethylpropylamine) (0.021 mol; 4 g) in acetonitrile (10 mL) was added *n*-iodododecane (0.042 mol; 18.7 g) and sodium carbonate (0.021 mol; 2.23 g). The reaction mixture was heated under reflux for 28 hours. The solvent was evaporated under reduced pressure and the residue was dried over P_4O_{10} and then crystallized from acetonitrile. White solid, mp 174-176 °C, yield 85%.

^{1}H NMR (403 MHz, $CDCl_3$): δ 3.59-3.44 (m, 4H), 3.42-3.36 (m, 8H), 3.19 (s, 12H), 2.52 (m, 4H), 1.87 (m, 4H), 1.46 (m, 8H), 1.35 (s, 12 H), 0.91 (t, 6H),**^{13}C NMR (101 MHz, $CDCl_3$)** δ 66.10, 60.89, 50.36, 45.52, 31.50-29.91, 22.50, 19.96. 12.43

<u>Preparation of compound (20)</u>

To a solution of 3,3′-Iminobis(*N*,*N*-dimethylpropylamine) (0.021 mol; 4 g) in acetonitrile (10 mL) was added *n*-iodohexadecane (0.042 mol; 14.9 g) and sodium carbonate (0.021 mol; 2.23 g). The reaction mixture was heated under reflux for 12 hours. The solvent was evaporated under reduced pressure and the residue was dried over P_4O_{10} and then crystallized from acetonitrile. White solid, mp 149-150 °C, yield 82%.

^{1}H NMR (403 MHz, $CDCl_3$): δ 3.57-3.49 (m, 4H), 3.46-3.35 (m, 8H), 3.19 (s, 12H), 2.54 (m, 4H), 1.87 (m, 4H), 1.46 (m, 8H), 1.35 (s, 28 H), 0.92 (t, 6H). **^{13}C NMR (101 MHz, $CDCl_3$)** δ 66.11, 60.89, 50.37, 45.52, 31.57-29.01, 22.51, 19.98. 12.14 **ESI-MS** (*m*/*z*) 323 $[C_{42}H_{99}N_3]^{2+}$

<u>Preparation of compound (21)</u>

To a solution of 3,3′-Iminobis(*N*,*N*-dimethylpropylamine) (0.021 mol; 4 g) in acetonitrile (10 mL) was added *n*-iodooctadecane (0.042 mol; 15,96 g) and sodium carbonate (0.021 mol; 2.23 g). The reaction mixture was heated under reflux for 7 hours. The solvent was evaporated under reduced pressure and the residue was dried over P_4O_{10} and then crystallized from acetonitrile. White solid, mp 127-128 °C, yield 80%.

^{1}H–NMR (403 MHz, TFA-d): δ 3.57-3.55 (m, 4H), 3.46-3.42 (m, 8H), 3.19 (s, 12H), 2.54 (m, 4H), 1.87 (m, 4H), 1.46 (m, 8H), 1.35 (s, 28 H), 0.92 (t, 6H). **^{13}C NMR (101 MHz, TFA-d)** δ 66.08, 60.89, 50.89, 45.53, 31.59-25.71, 22.13, 19.94. 12.46 **ESI-MS** (*m*/*z*) 347 $[C_{46}H_{99}N_3]^{2+}$, 440 $[C_{28}H_{62}N_3]$

Compound 1 ^{1}H NMR

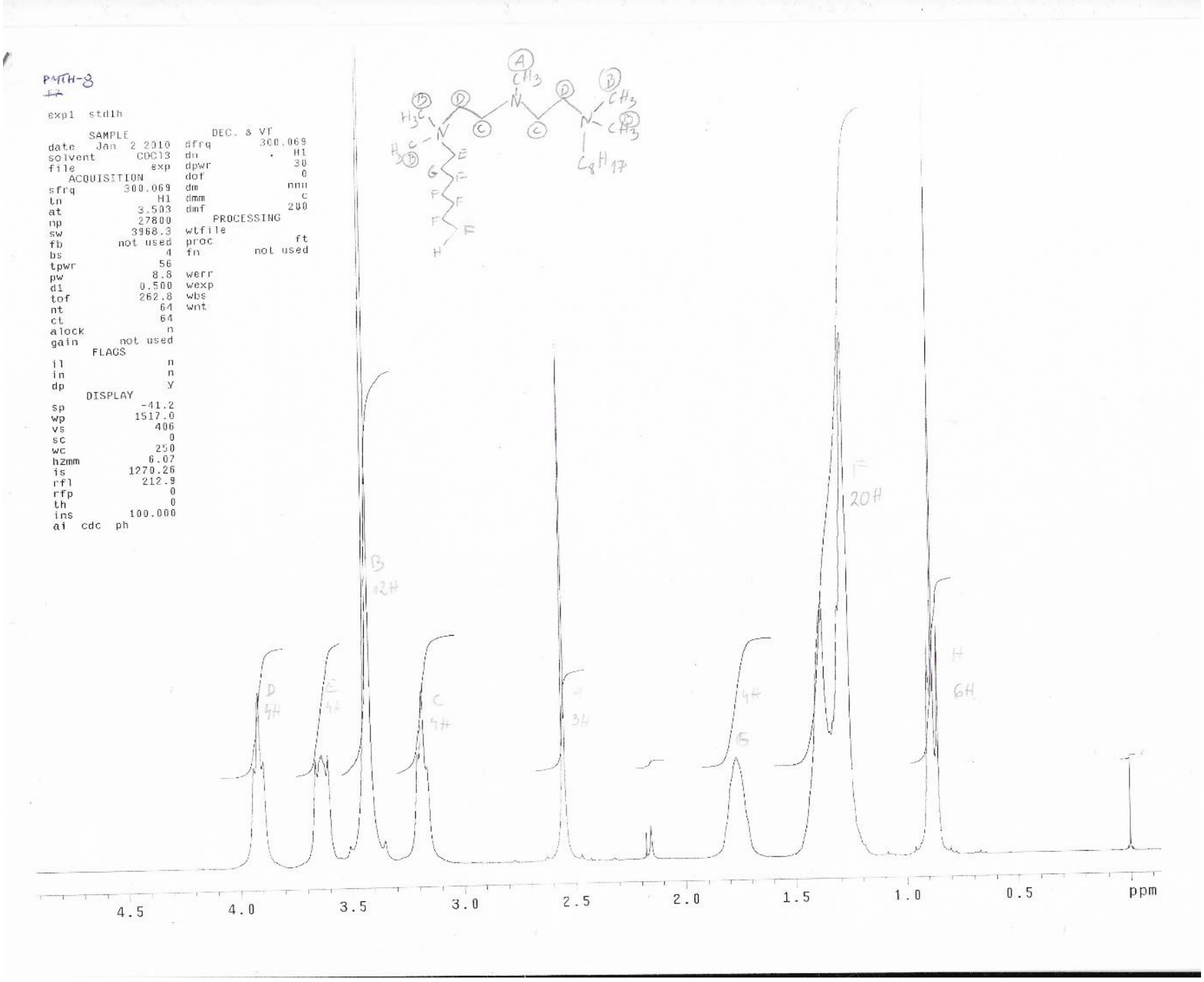

Compound 1 ^{13}C NMR

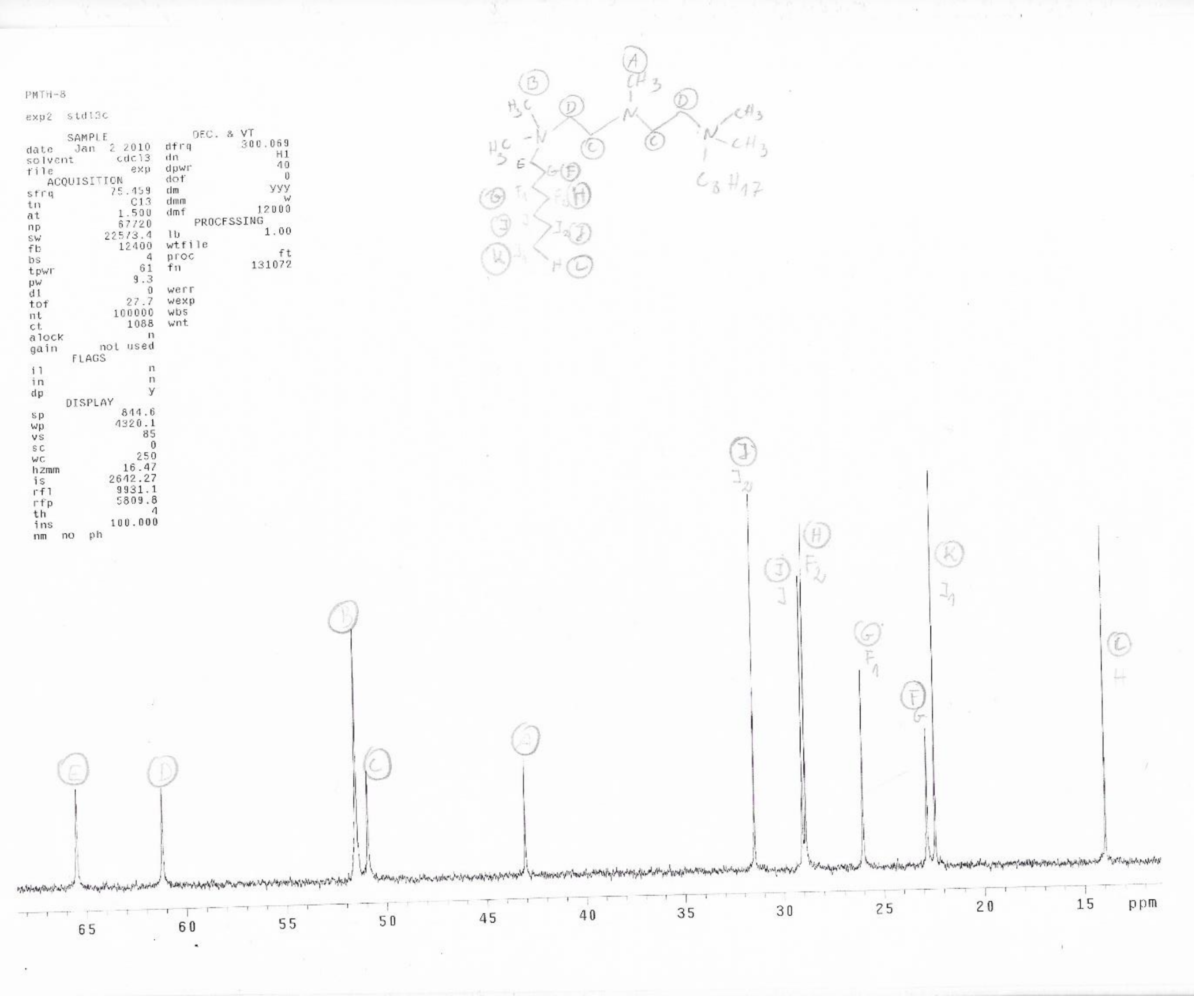

Compound 2 ^{1}H NMR

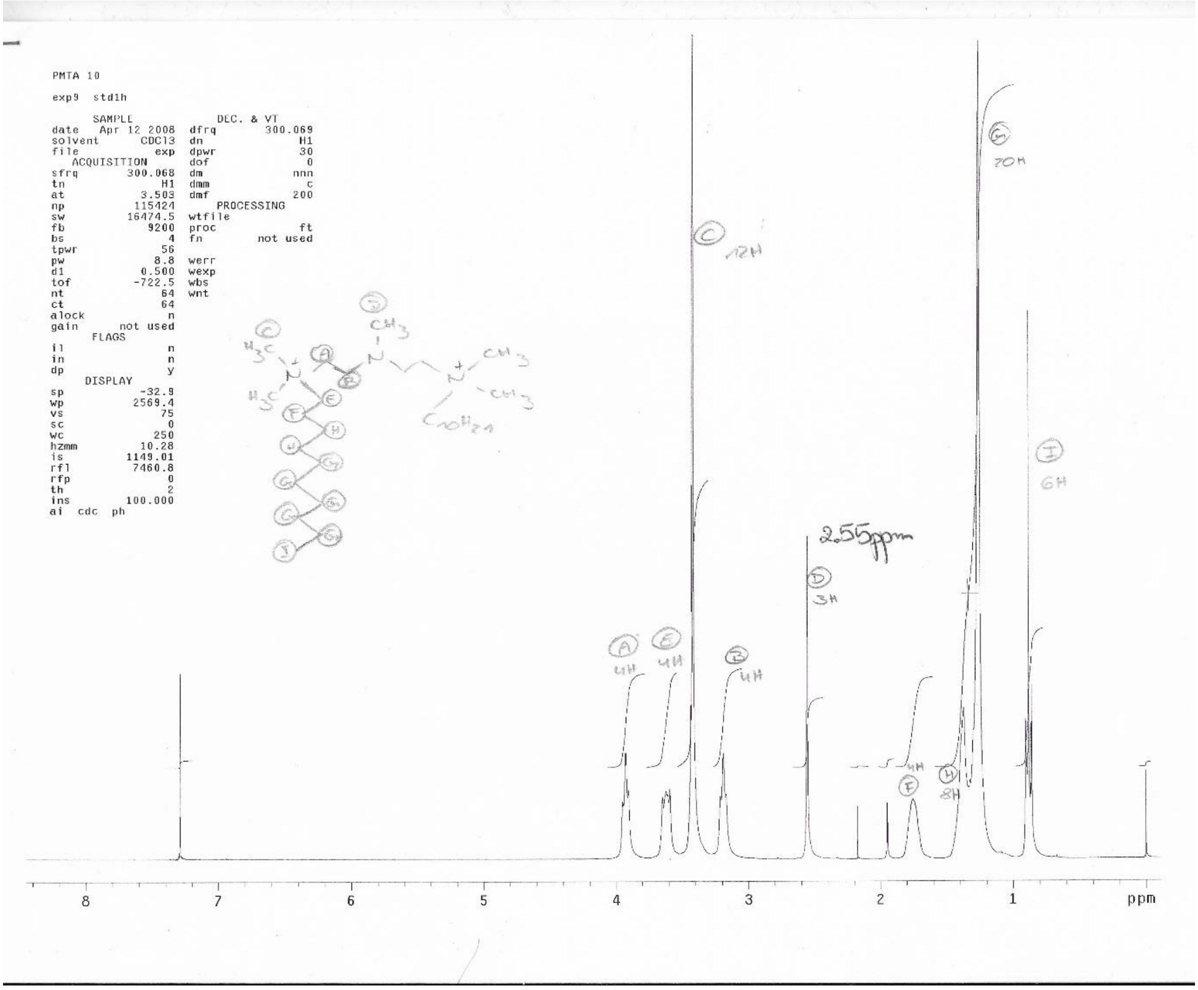

Compound 2 ^{13}C NMR

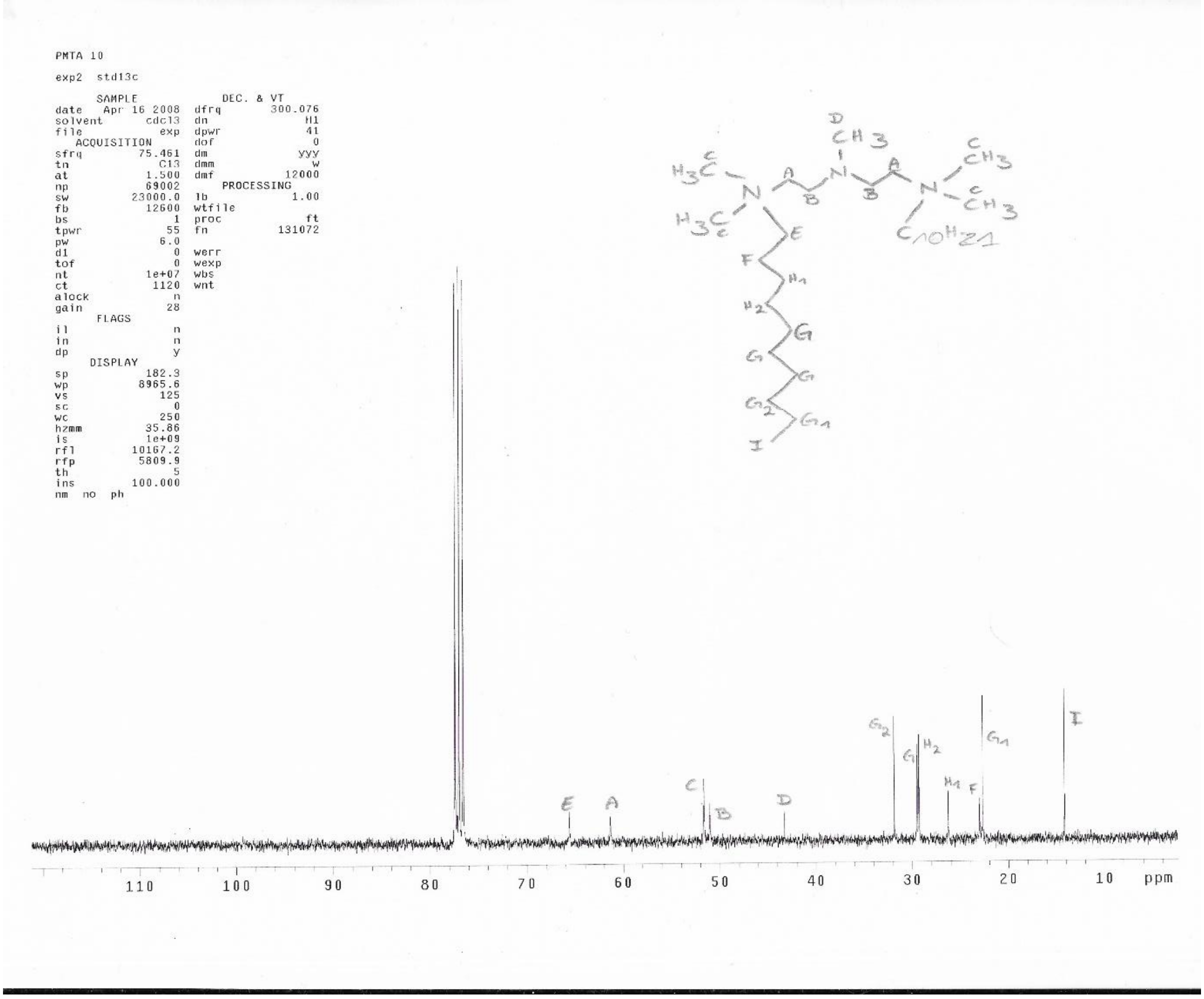

Compound 3 ^{1}H NMR

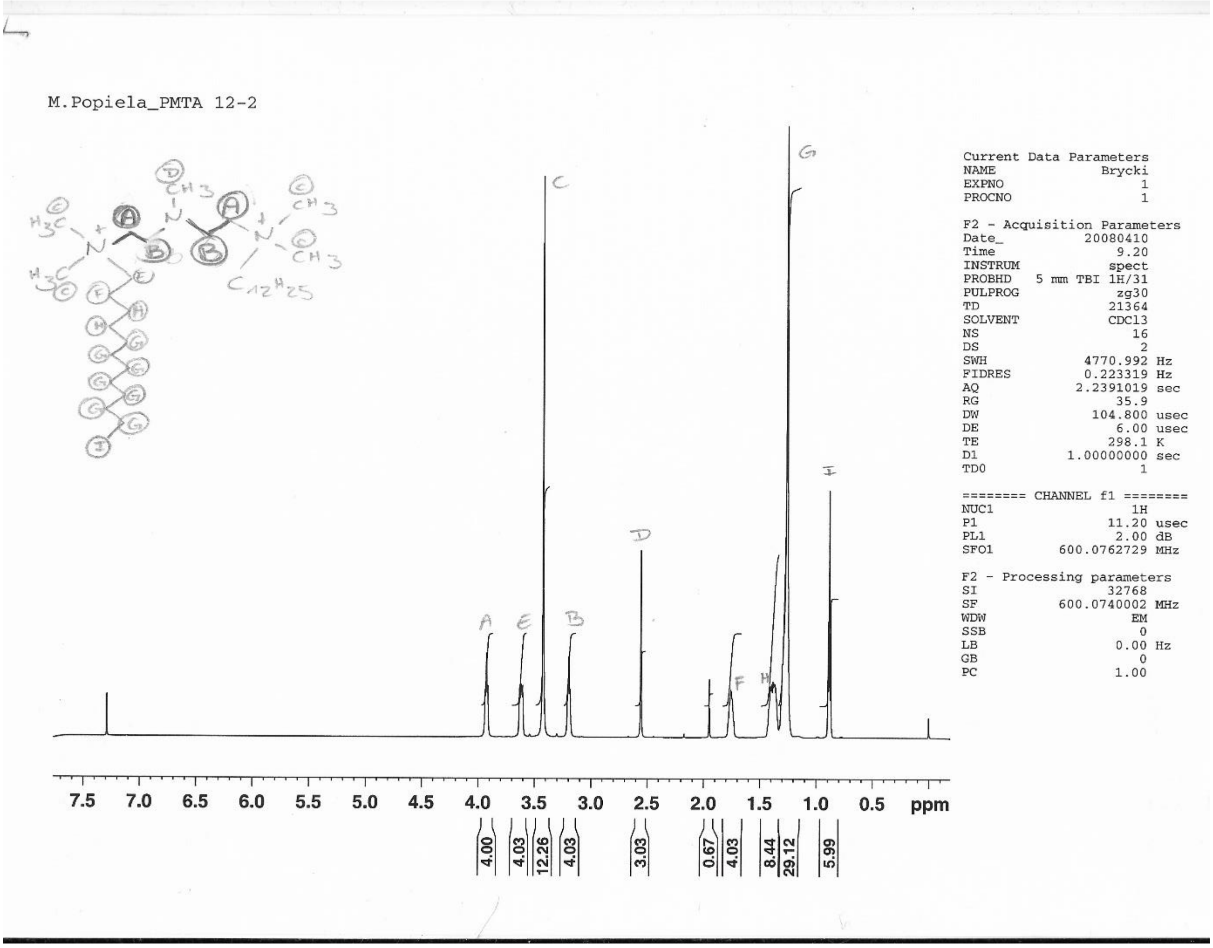

Compound 3 ¹³C NMR

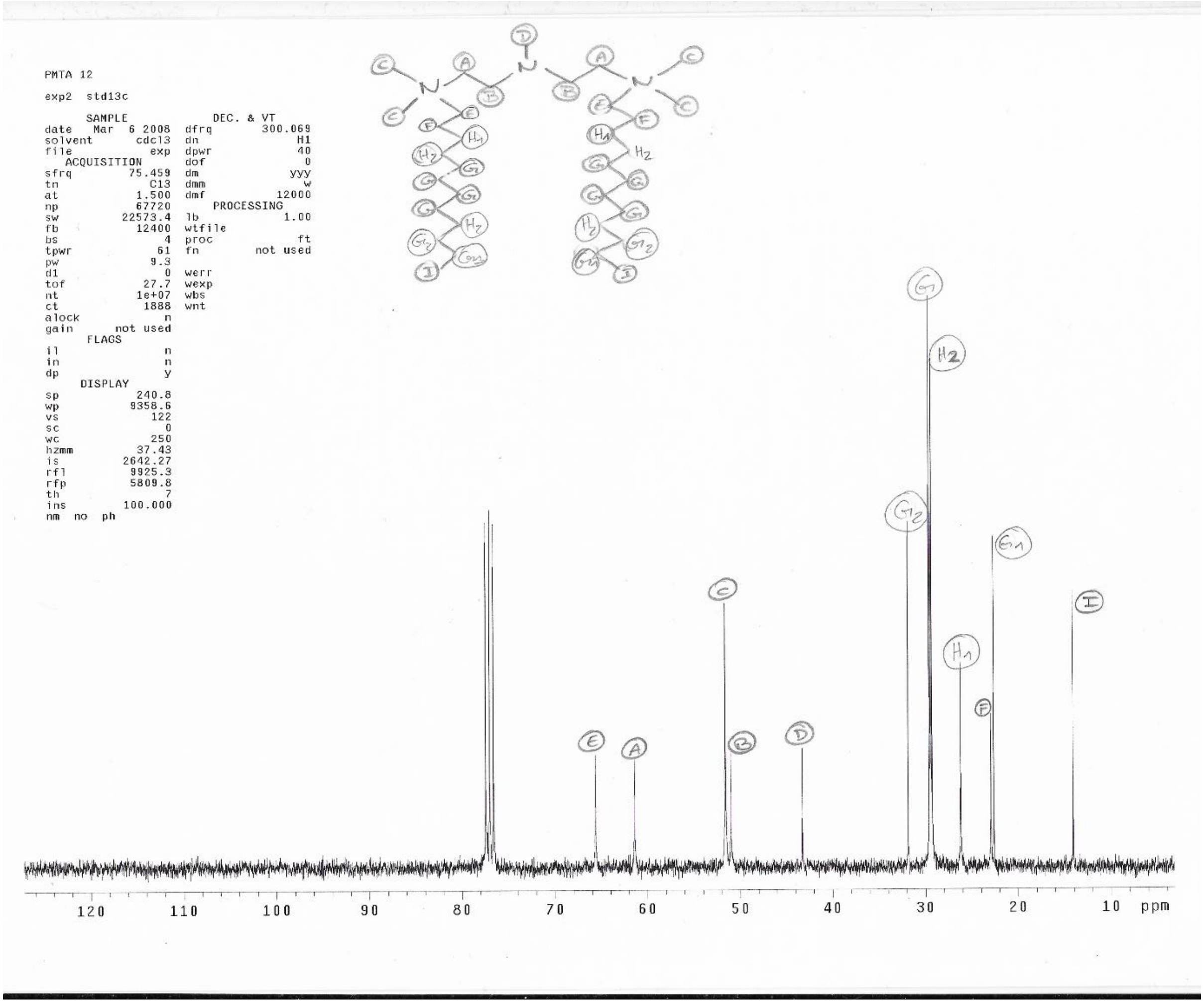

Compound 4 ^{1}H NMR

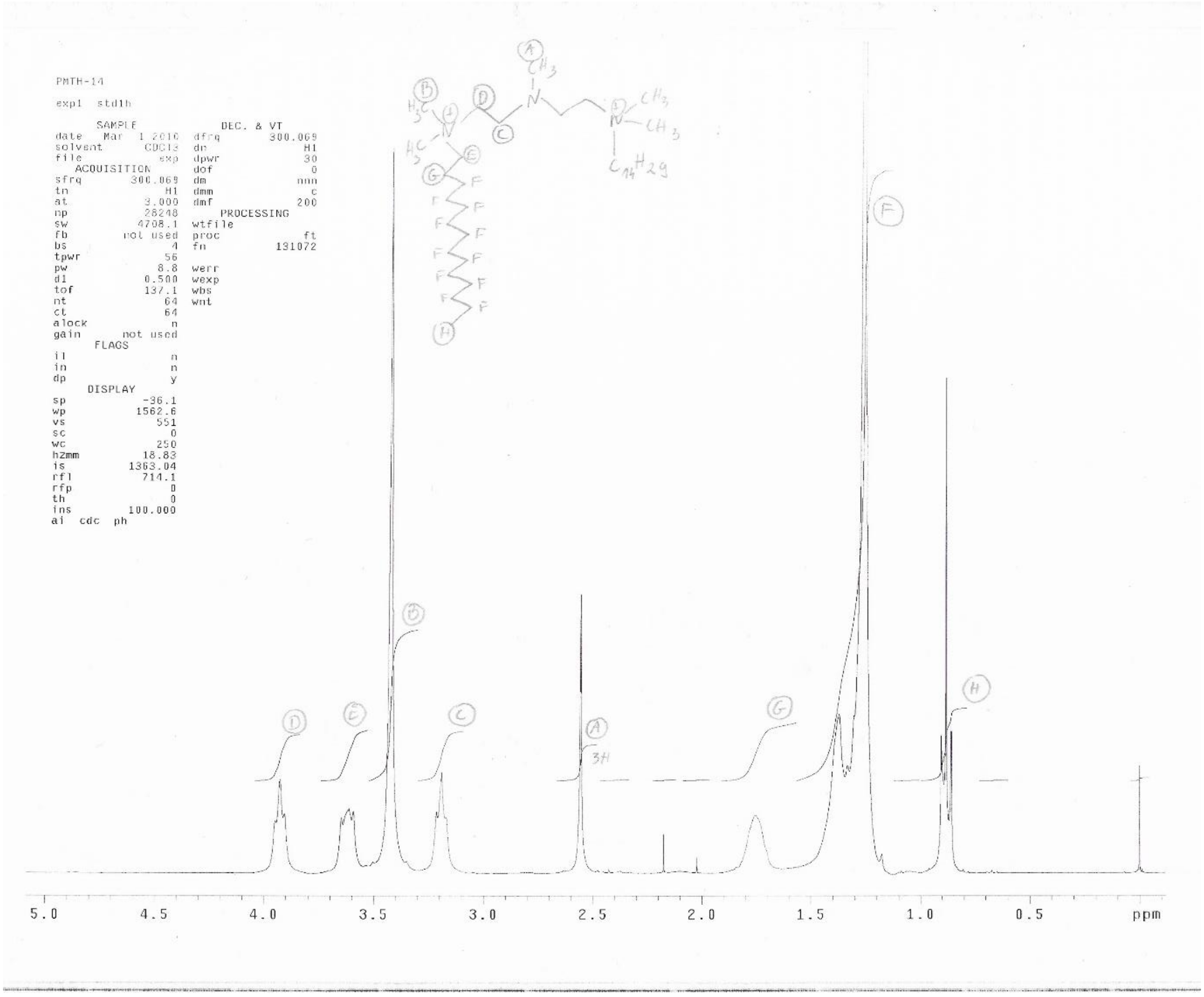

Compound 4 ^{13}C NMR

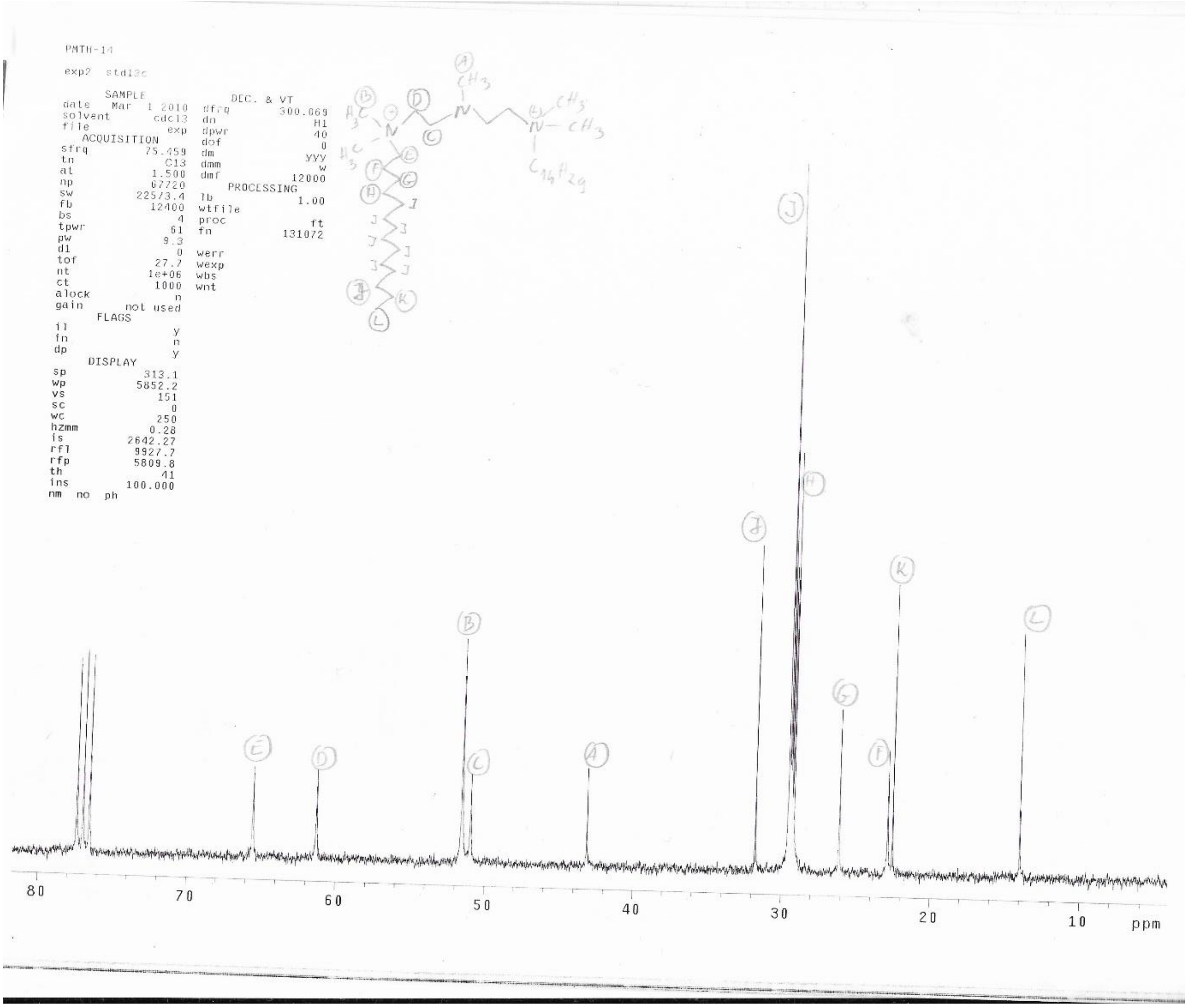

Compound 5 ^{1}H NMR

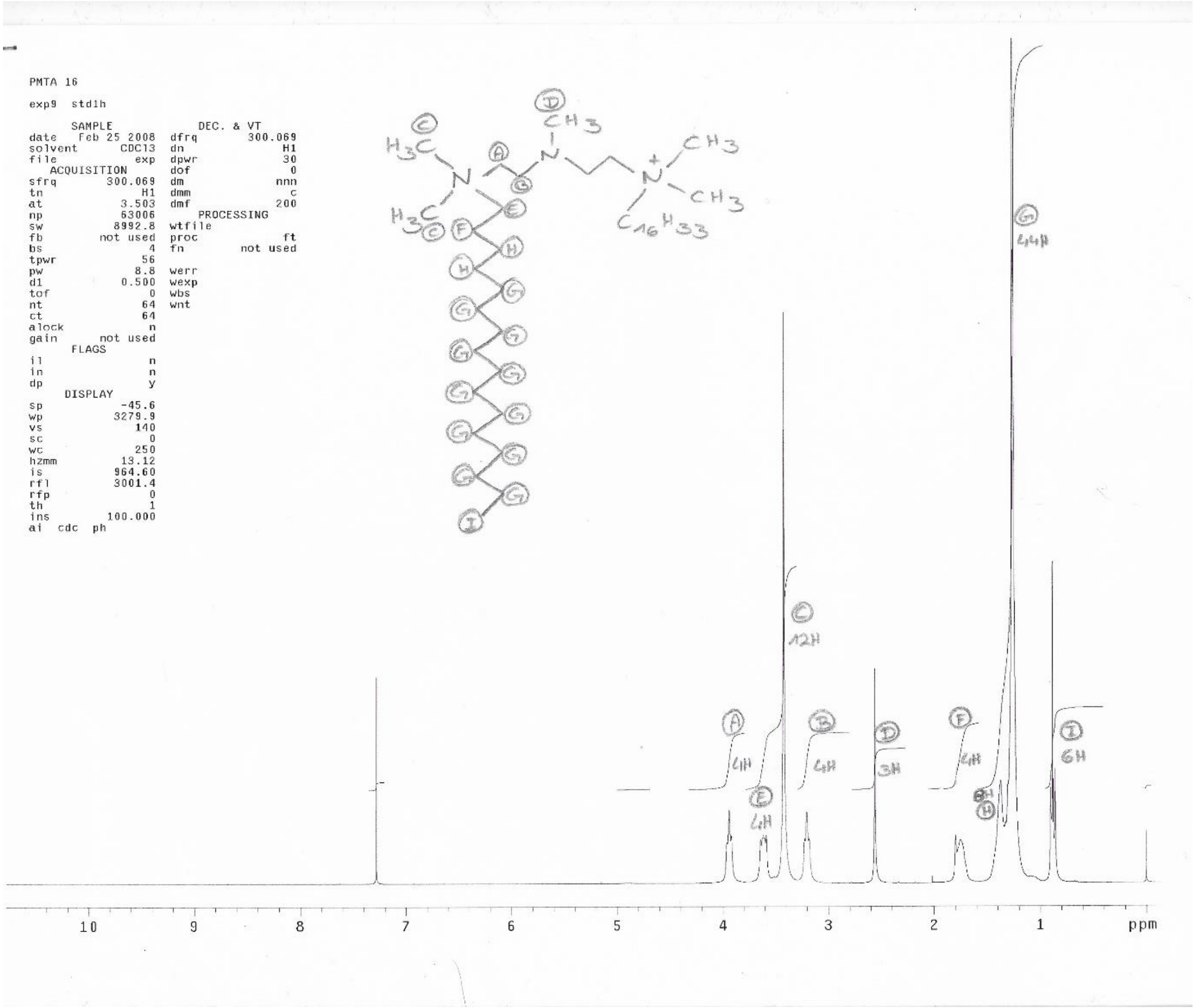

Compound 5 ^{13}C NMR

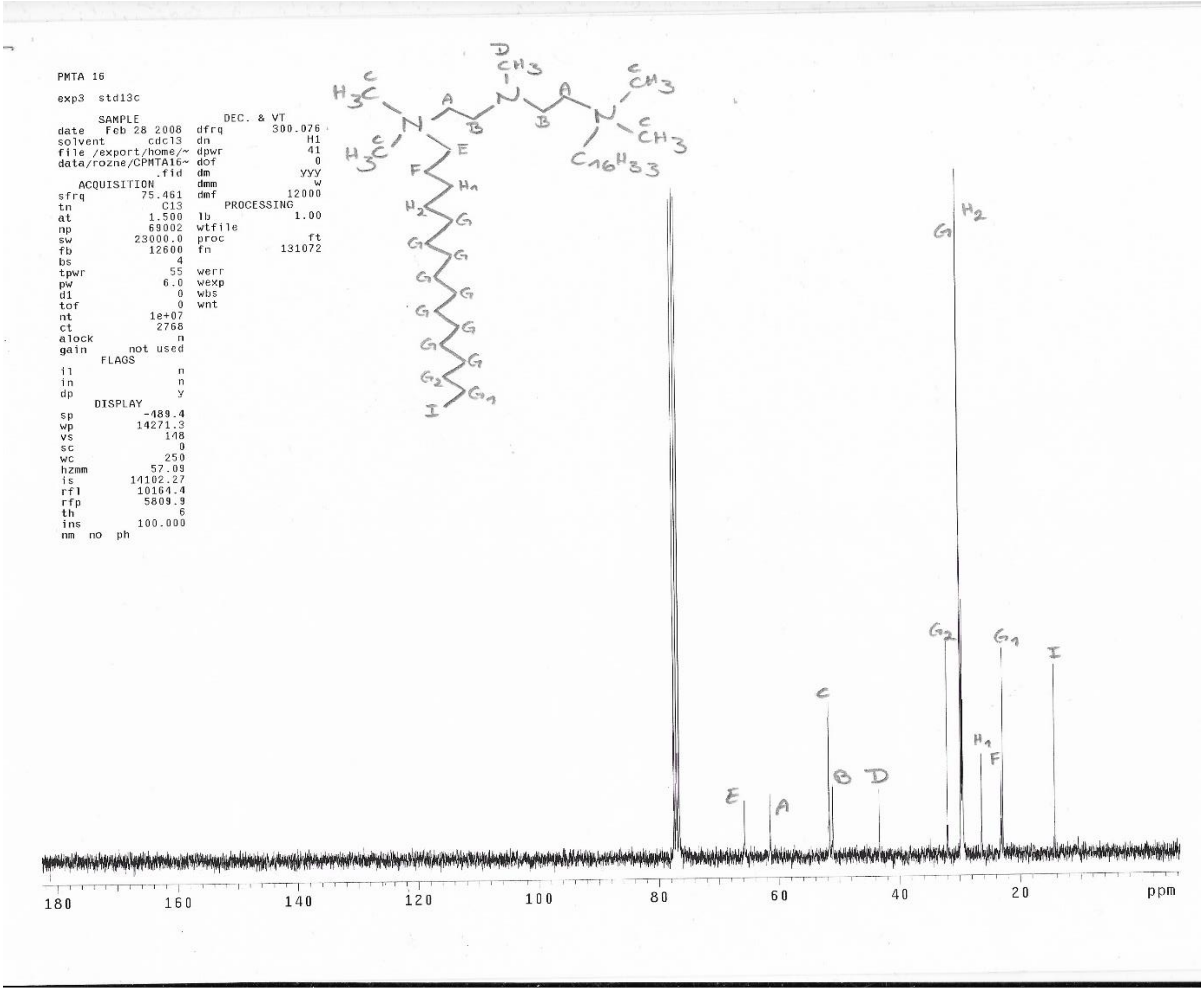

Compound 6 ^{1}H NMR

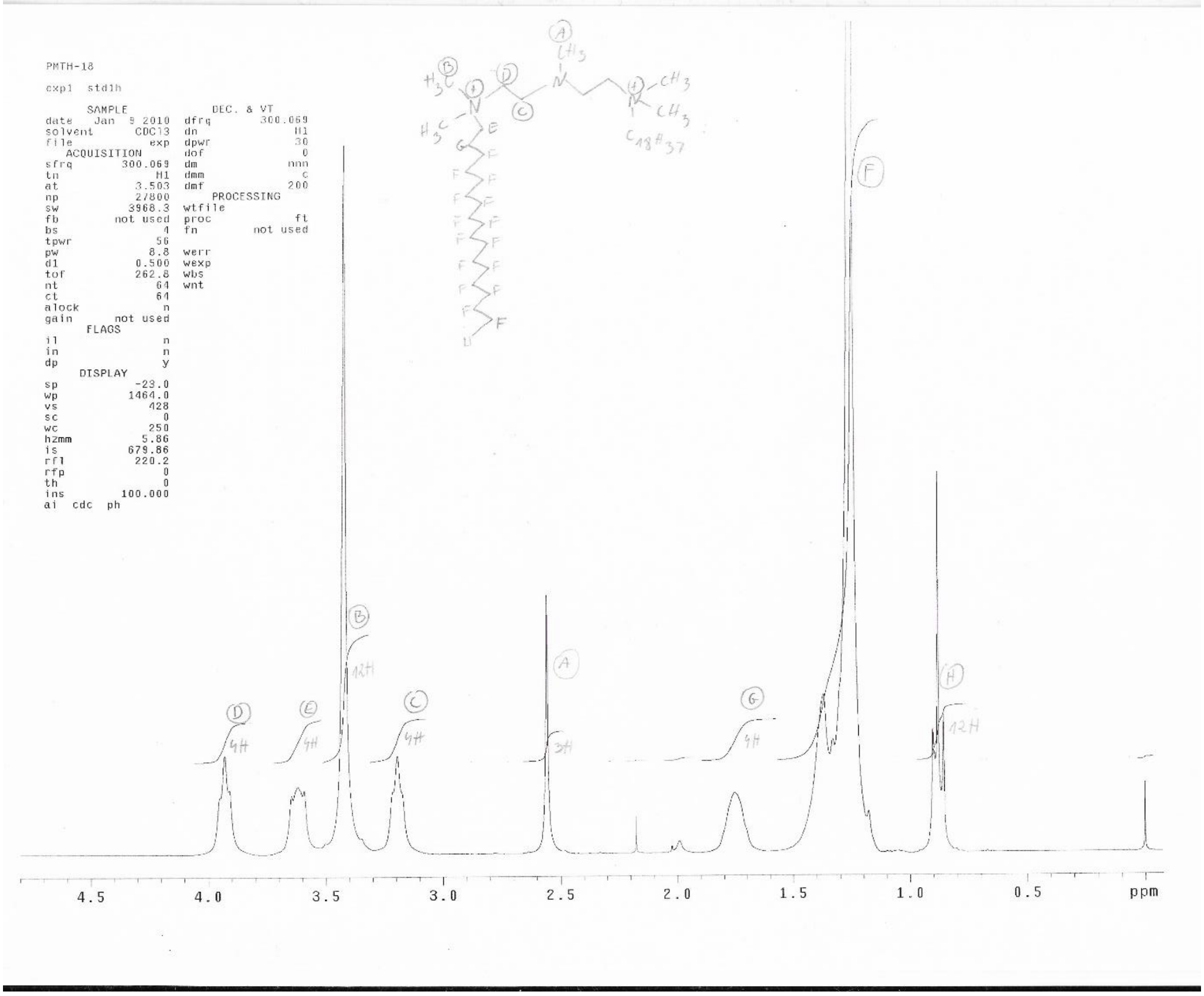

Compound 6 ^{13}C NMR

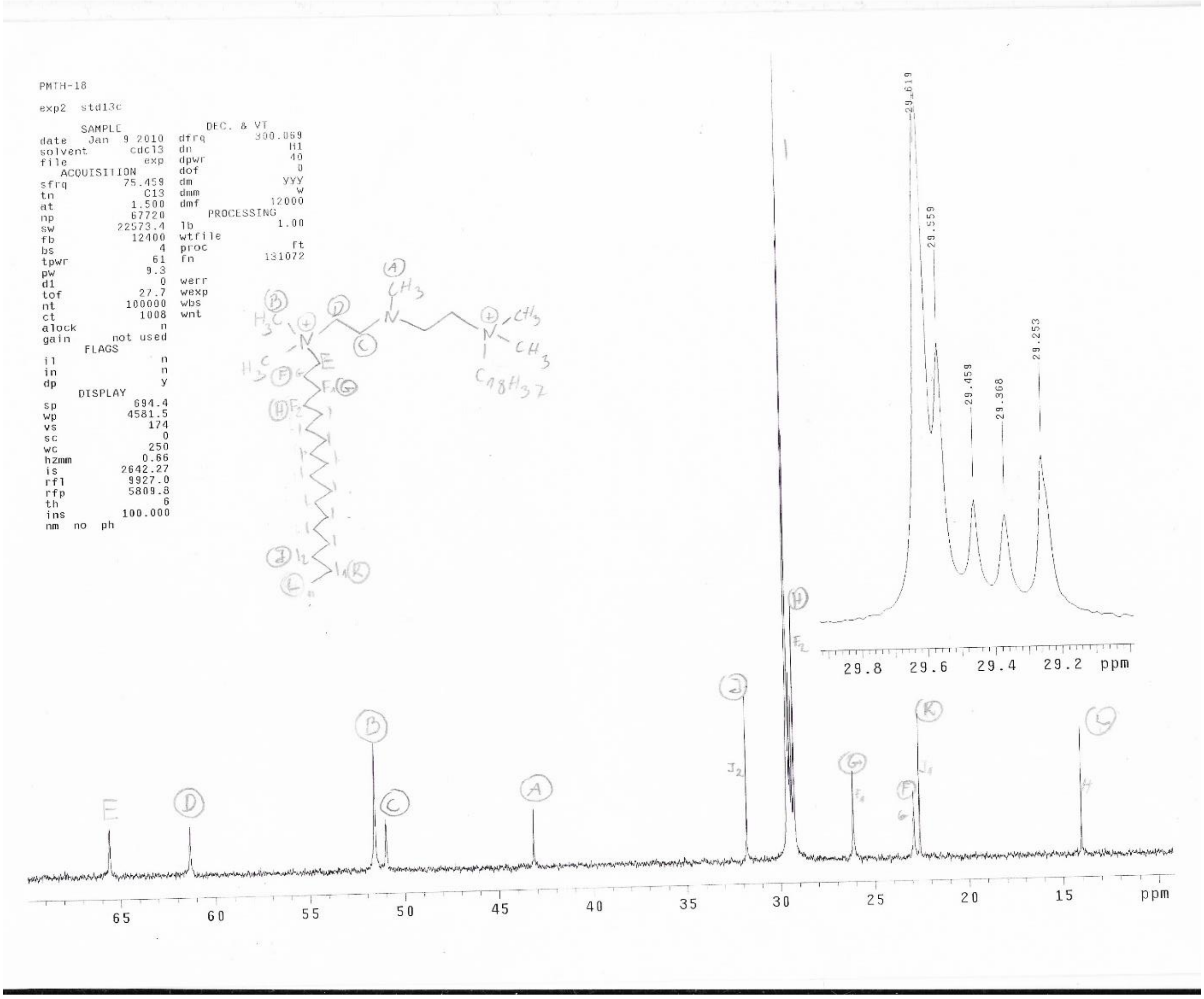

Compound 7 ^{1}H NMR

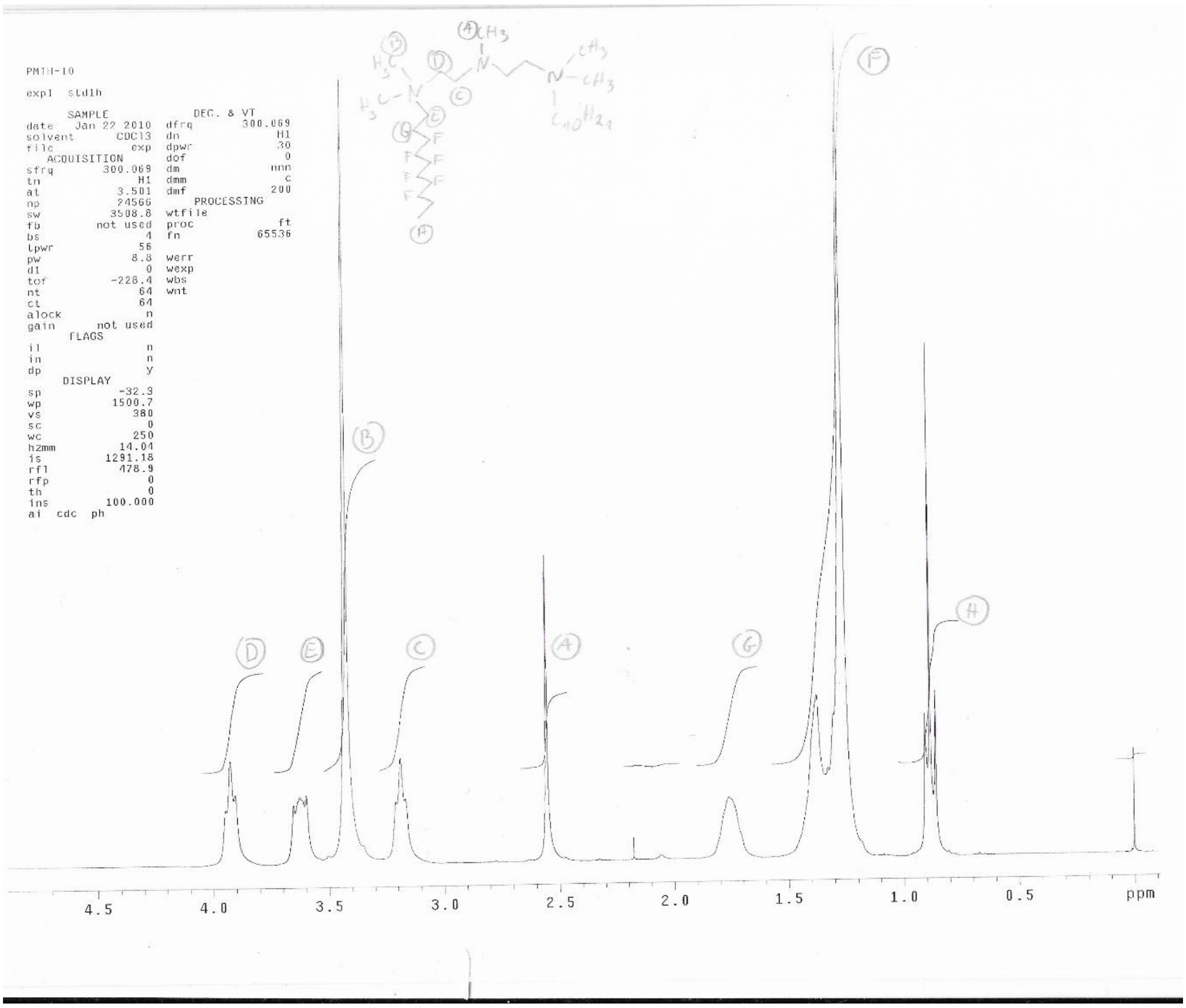

Compound 7 ^{13}C NMR

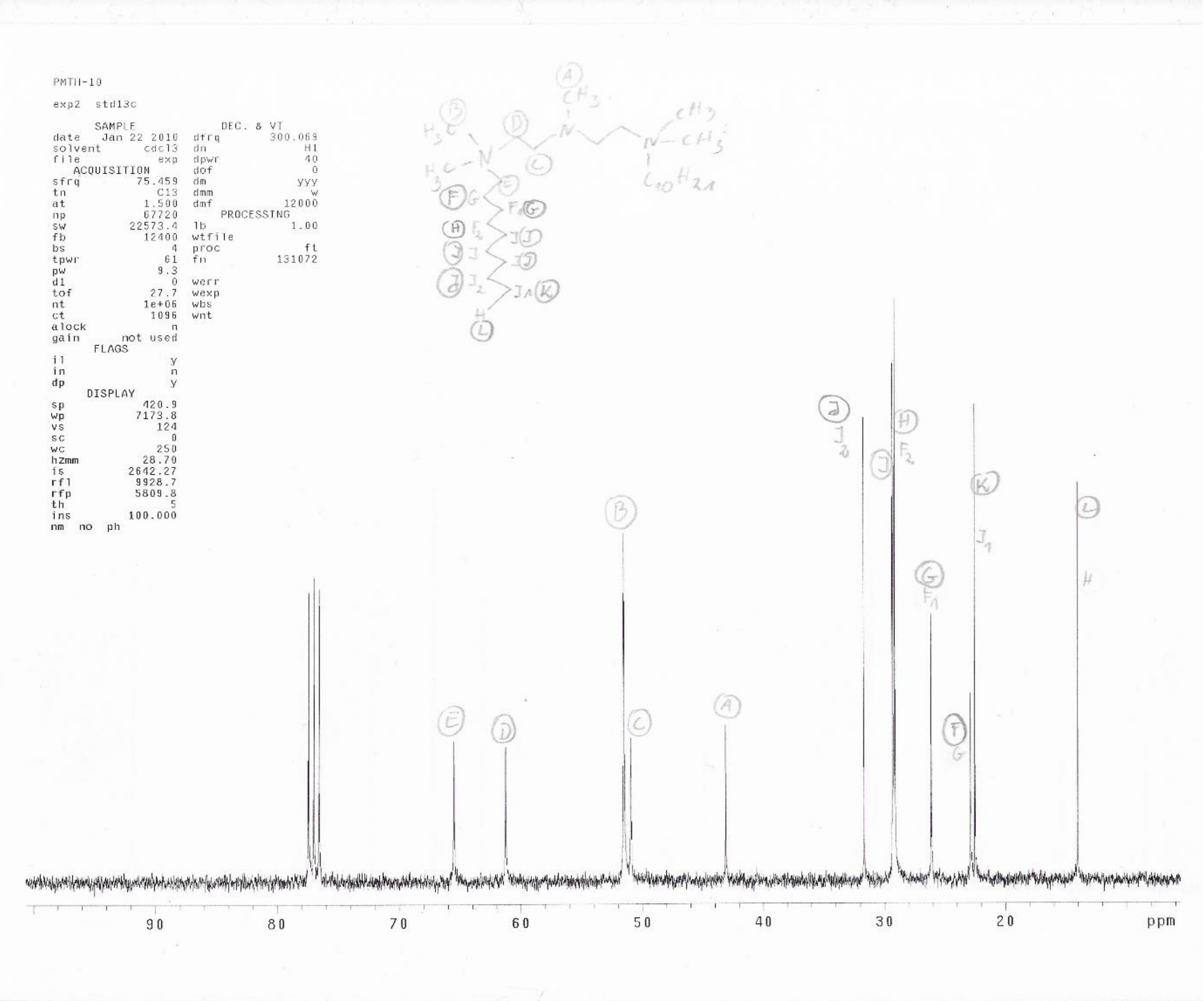

Compound 8 ¹H NMR

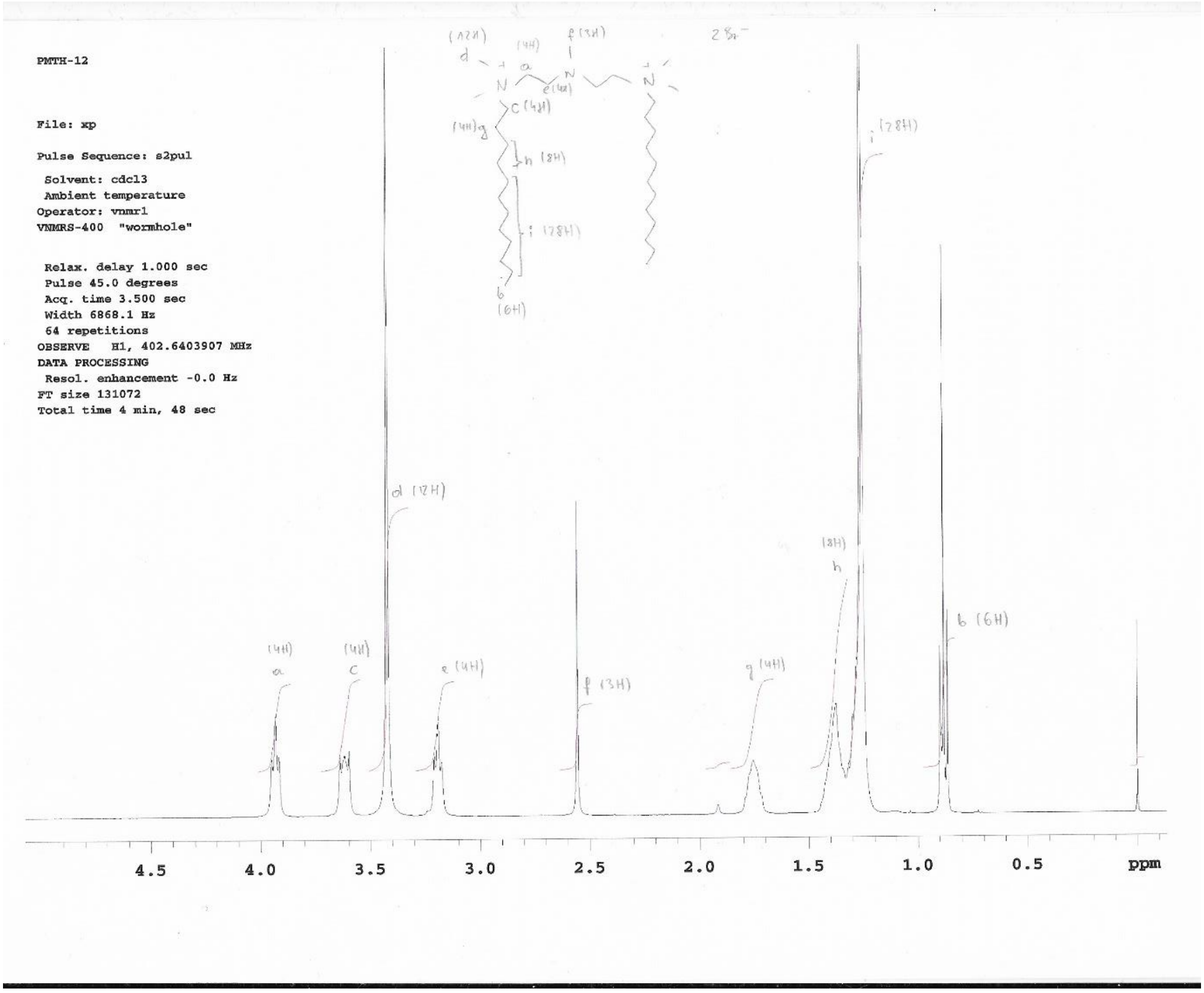

Compound 8 ^{13}C NMR

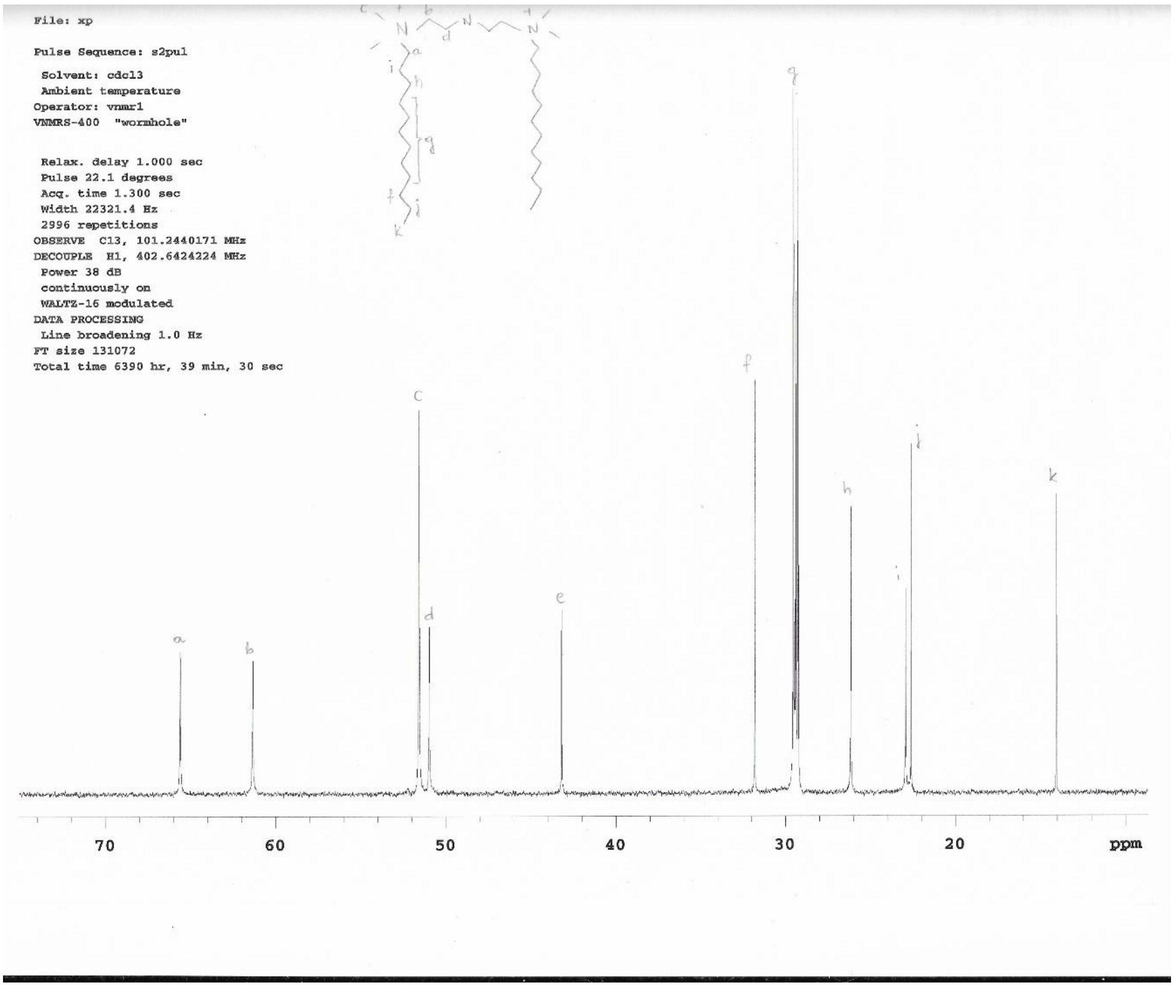

Compound 9 ¹H NMR

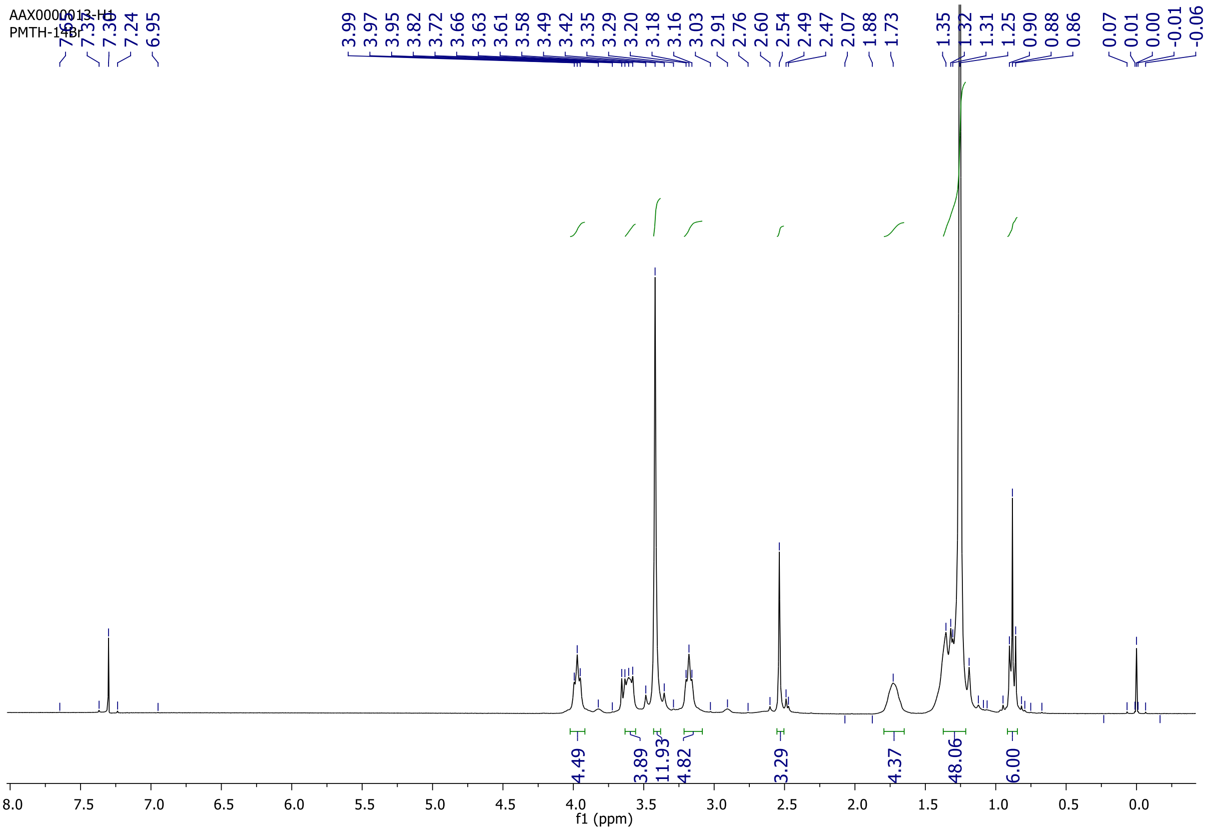

Compound 9 ^{13}C NMR

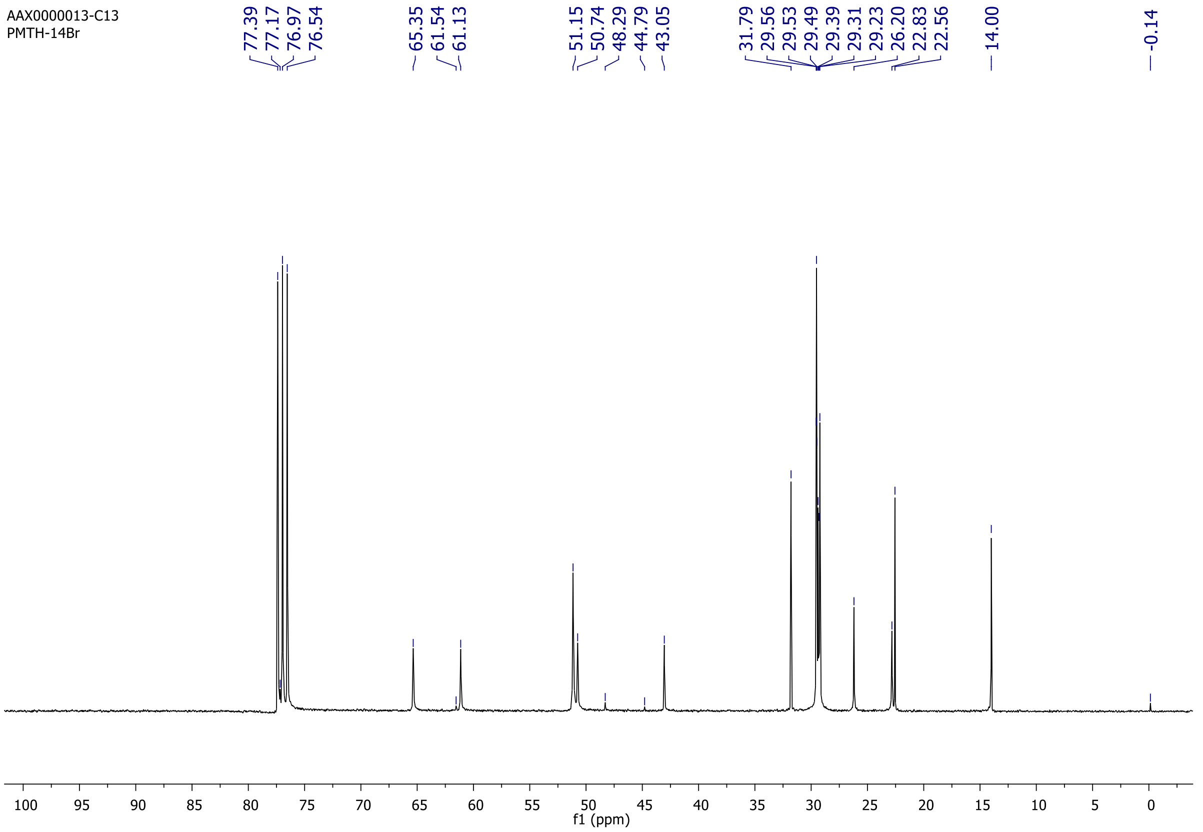

Compound 10 ^{1}H NMR

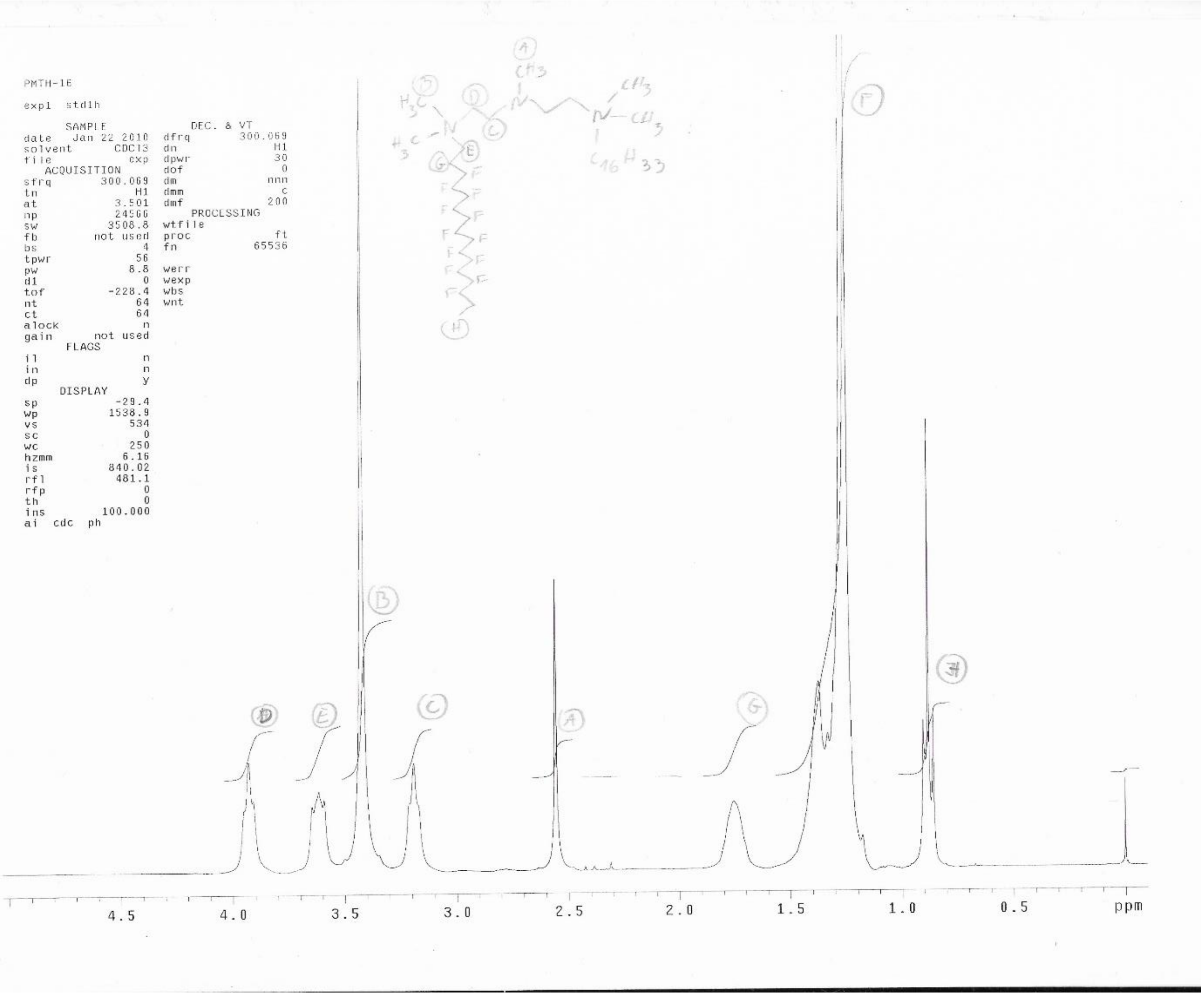

Compound 10 ^{13}C NMR

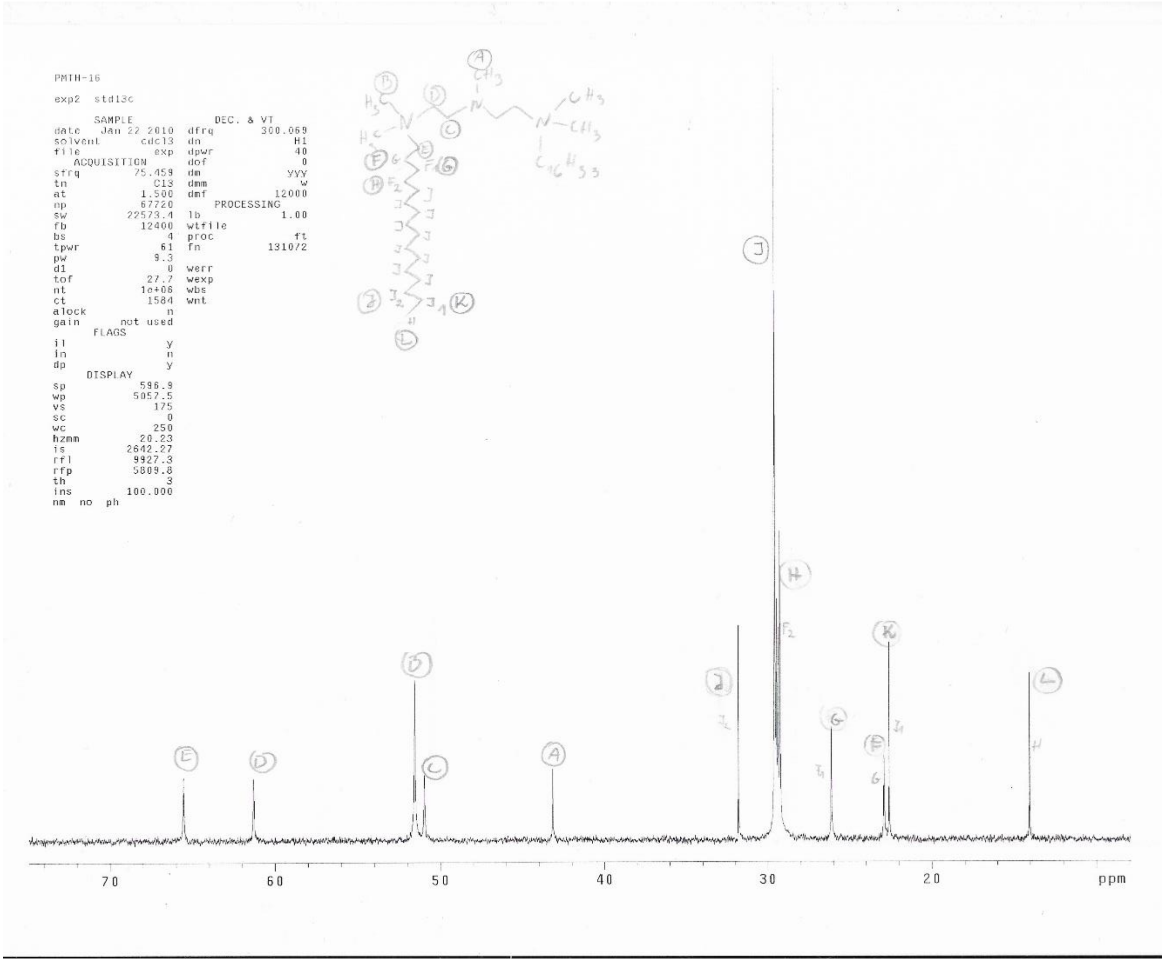

Compound 11 ^{1}H NMR

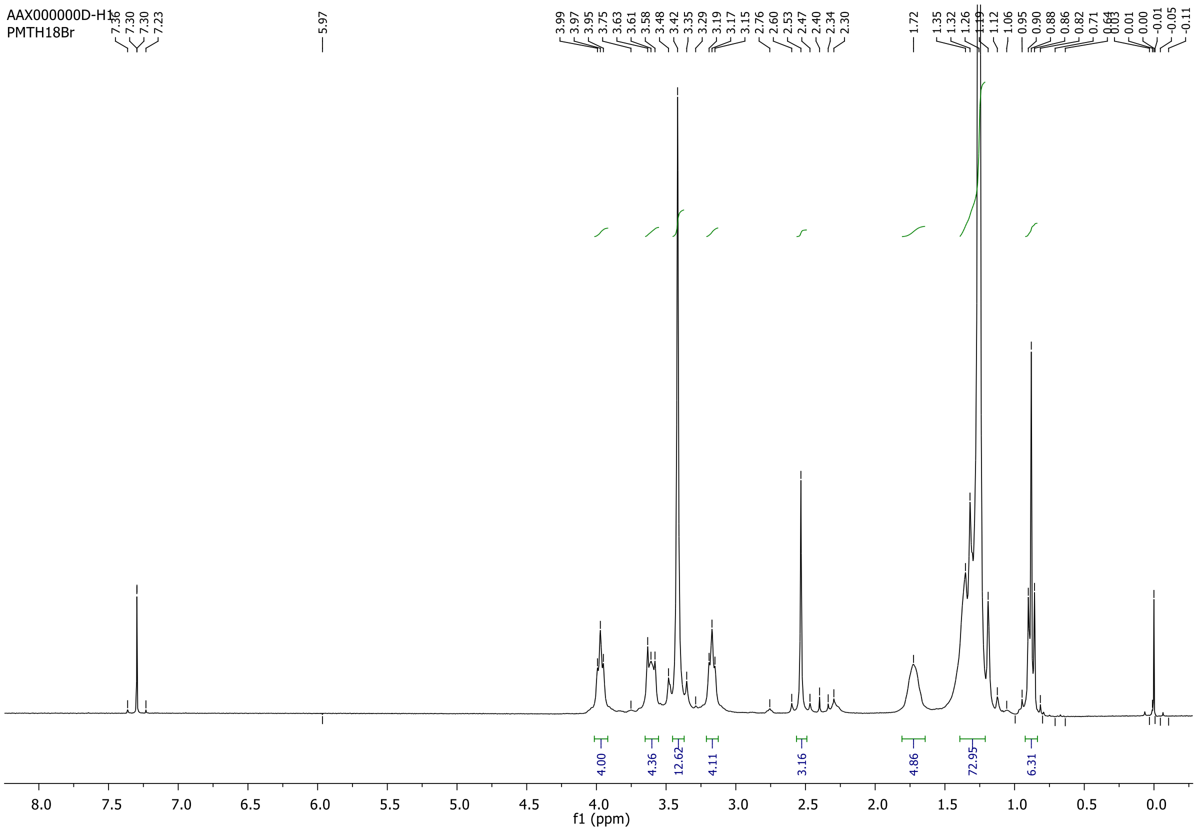

Compound 11 ^{13}C NMR

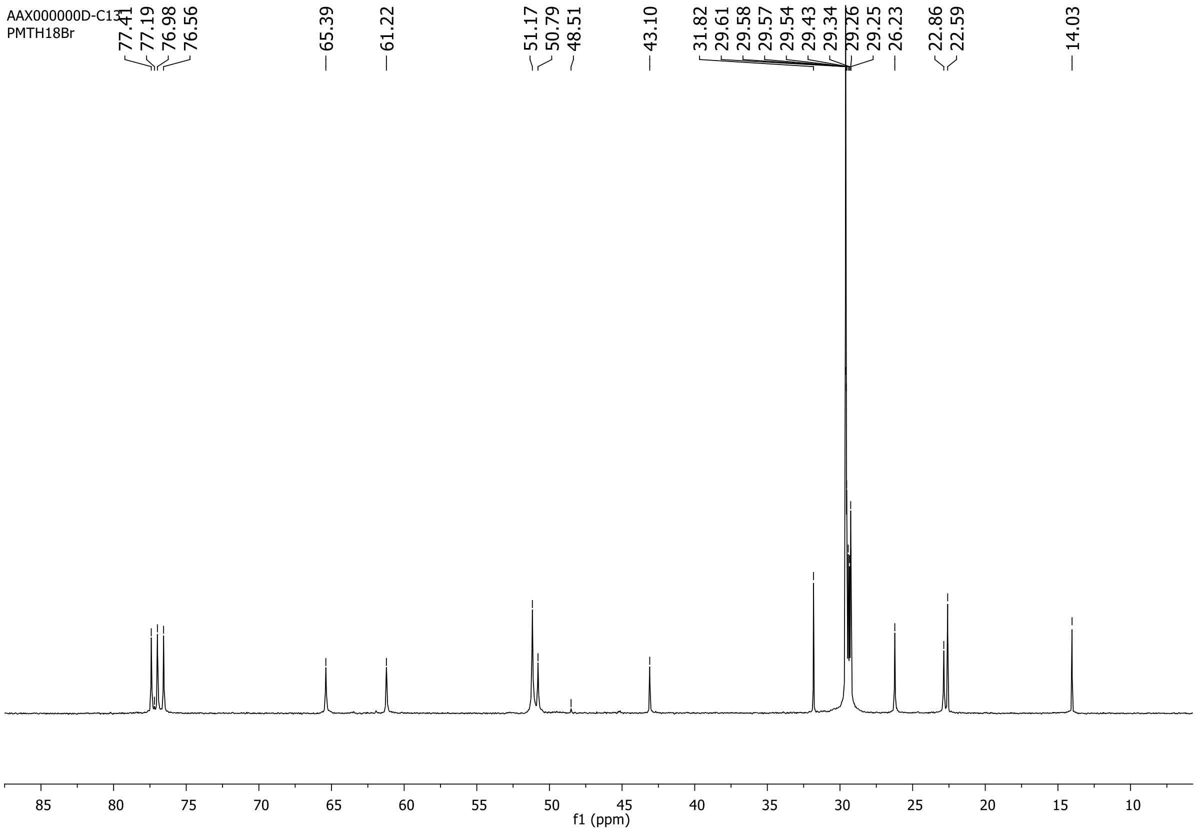

Compound 12 ^{1}H NMR

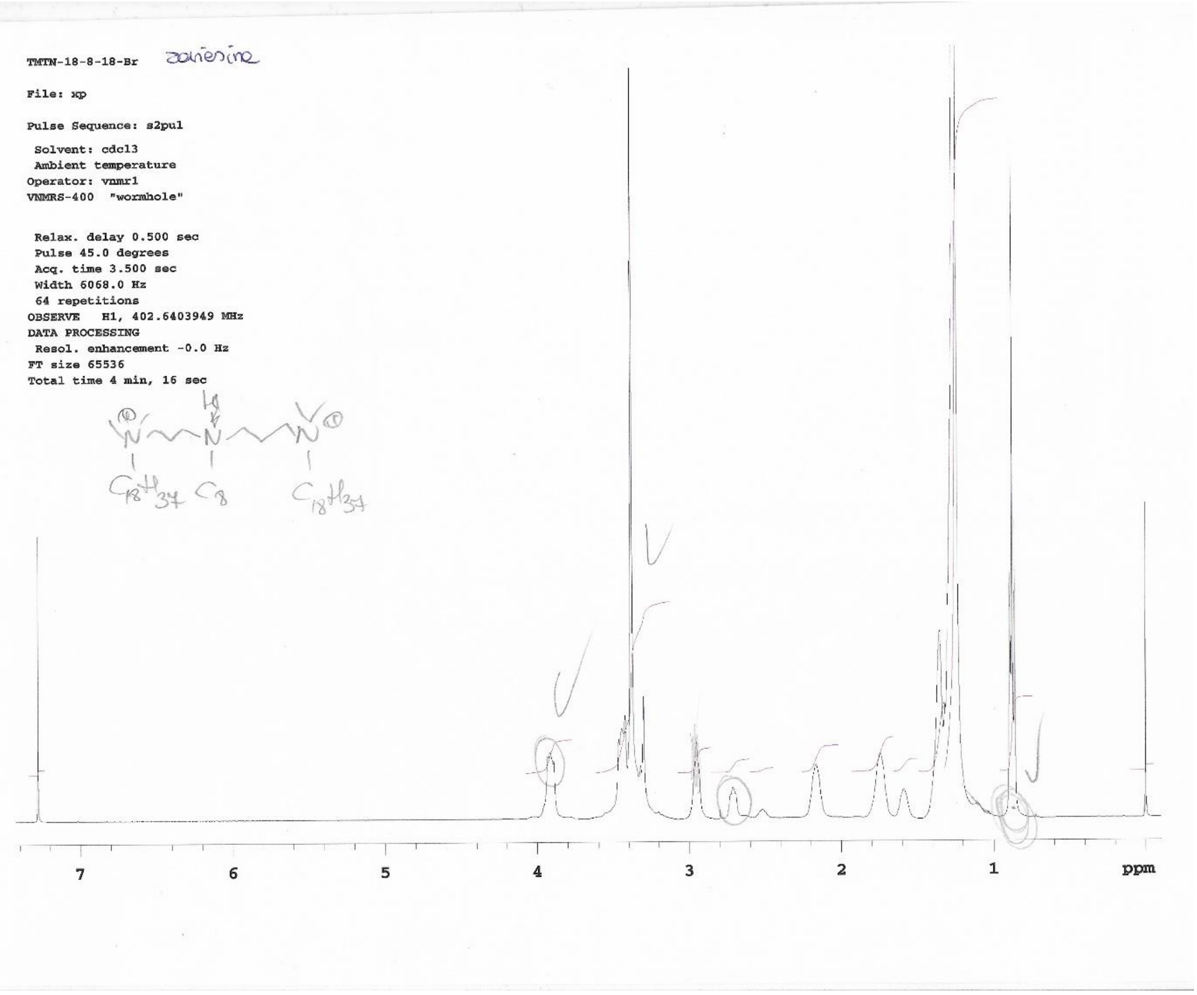

Compound 12 ^{13}C NMR

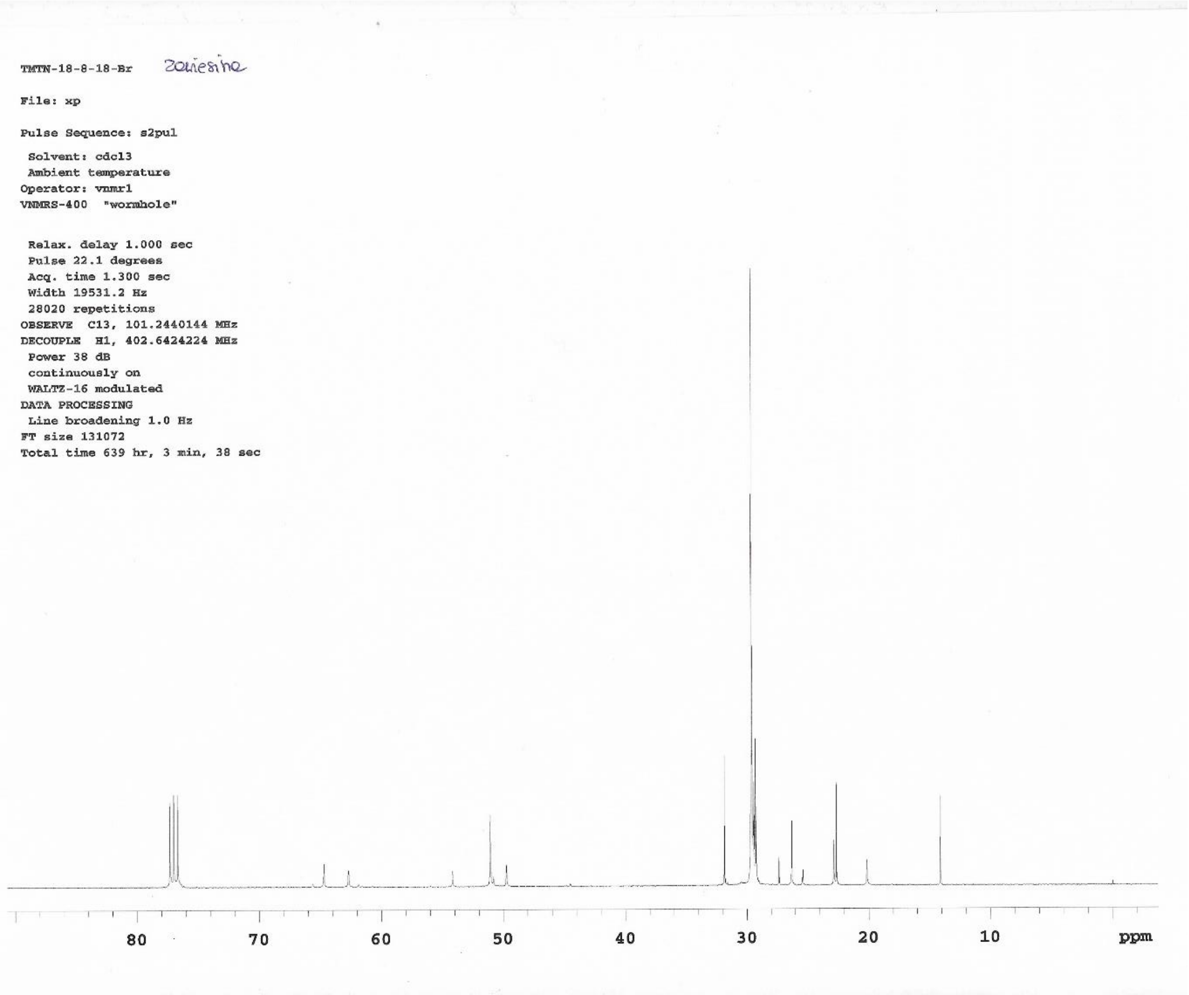

Compound 13 ^{1}H NMR

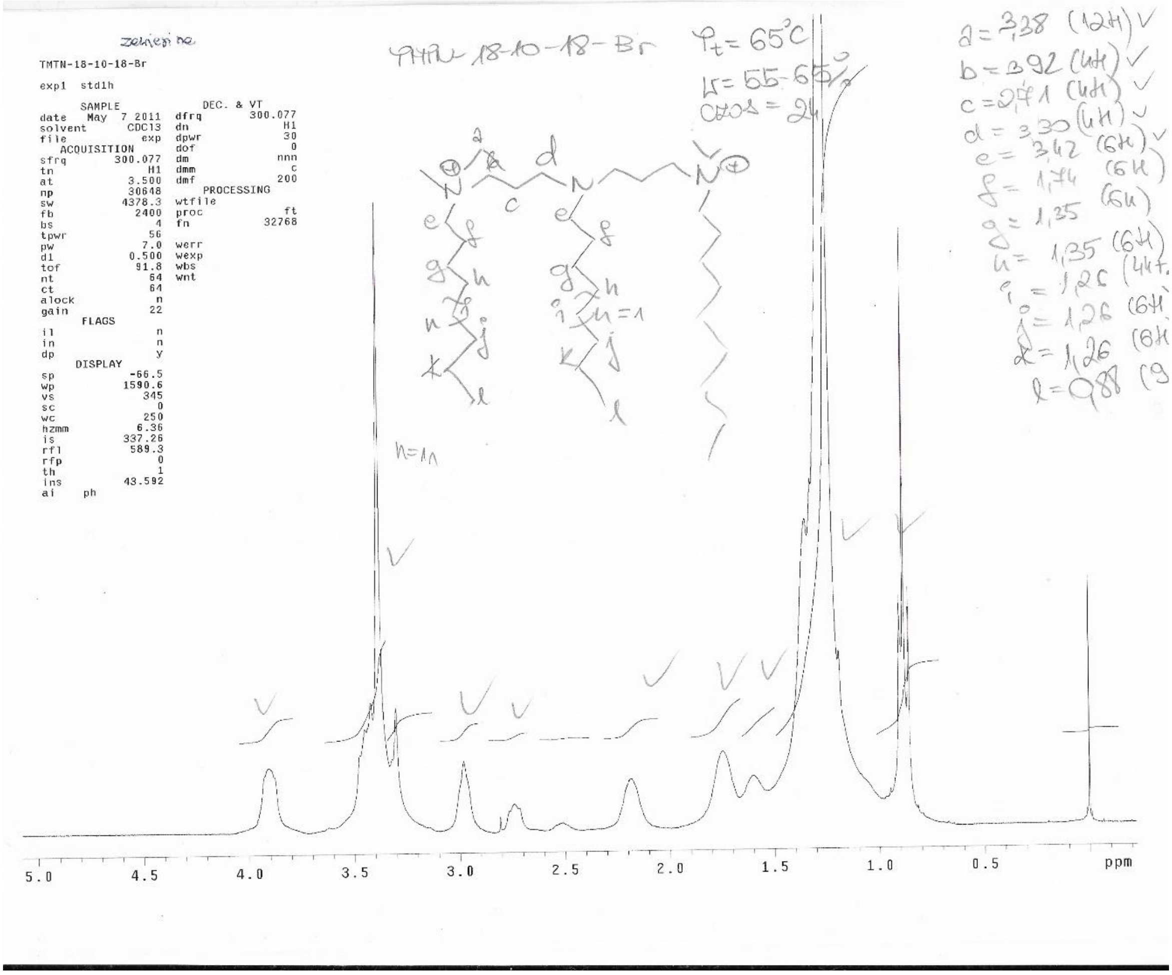

Compound 13 ^{13}C NMR

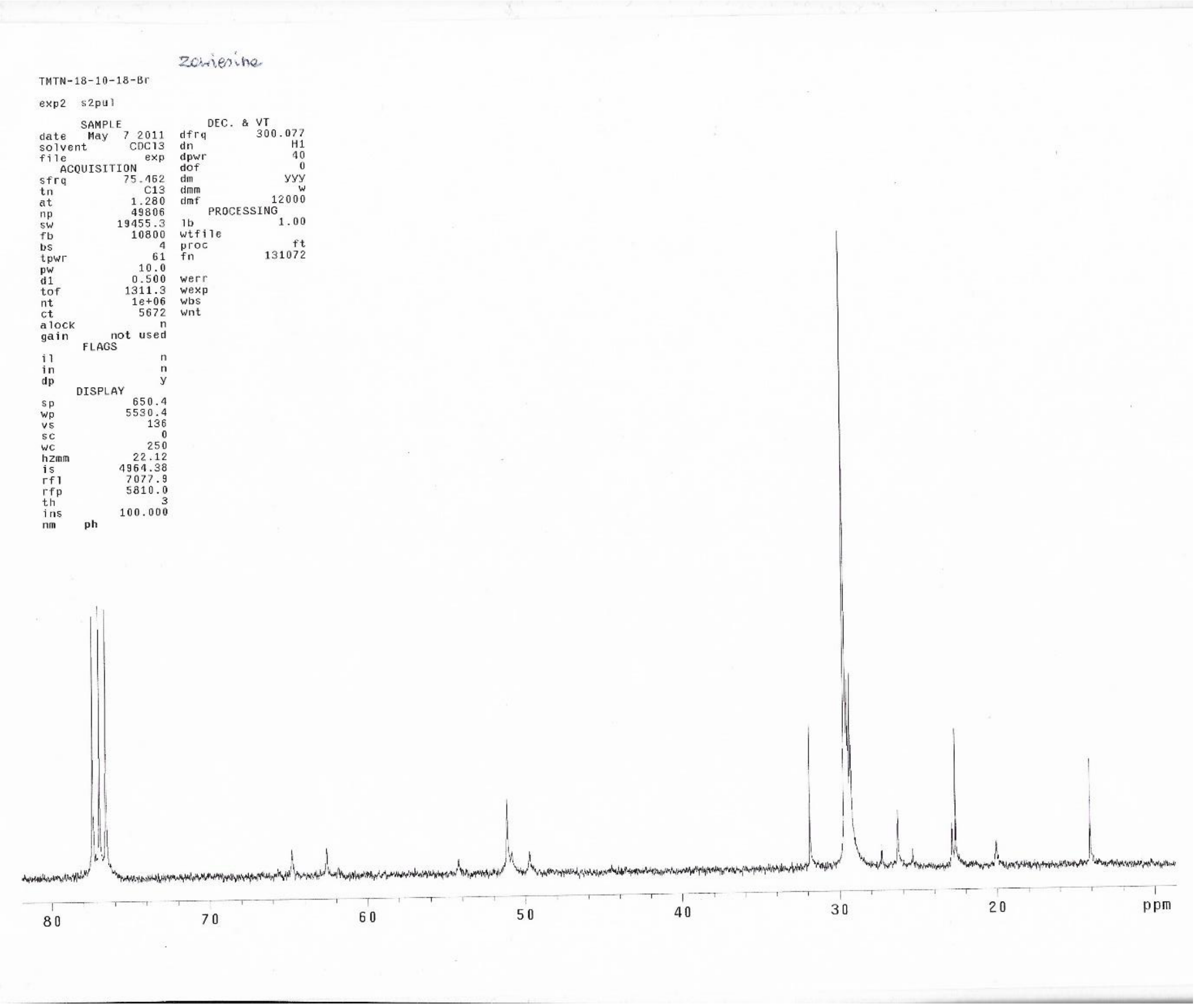

Compound 14 ^{1}H NMR

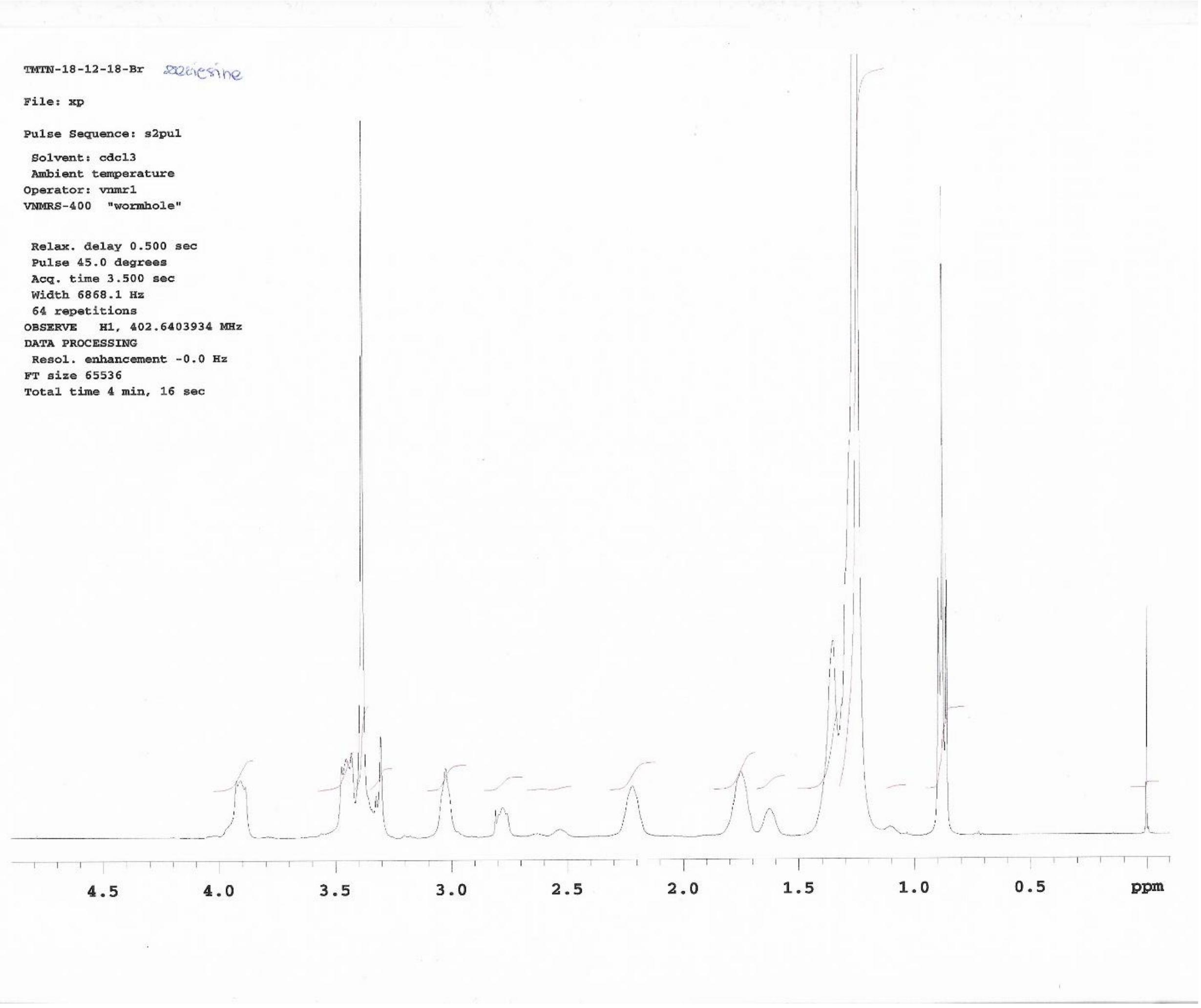

Compound 14 ^{13}C NMR

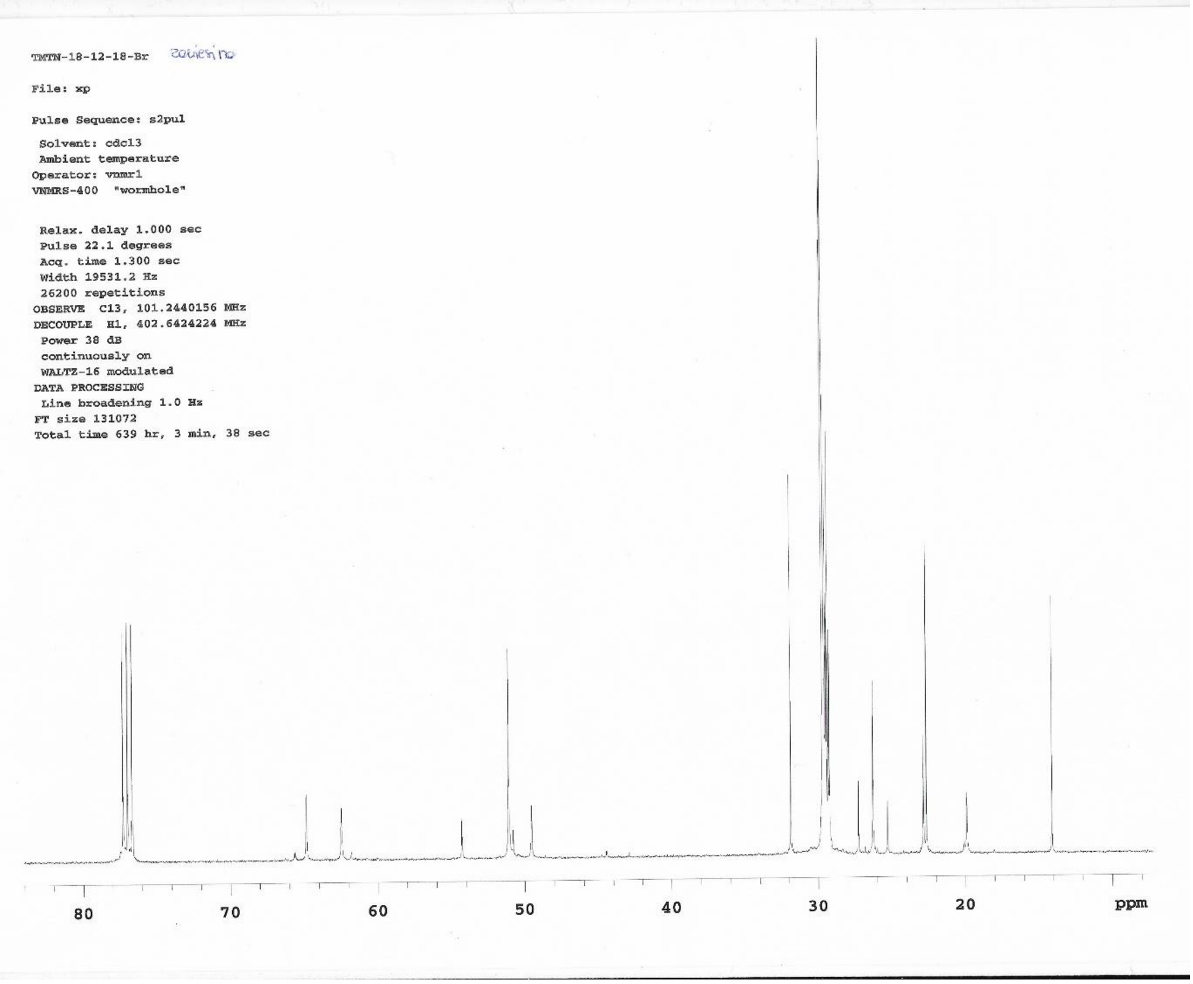

Compound 15 ¹H NMR

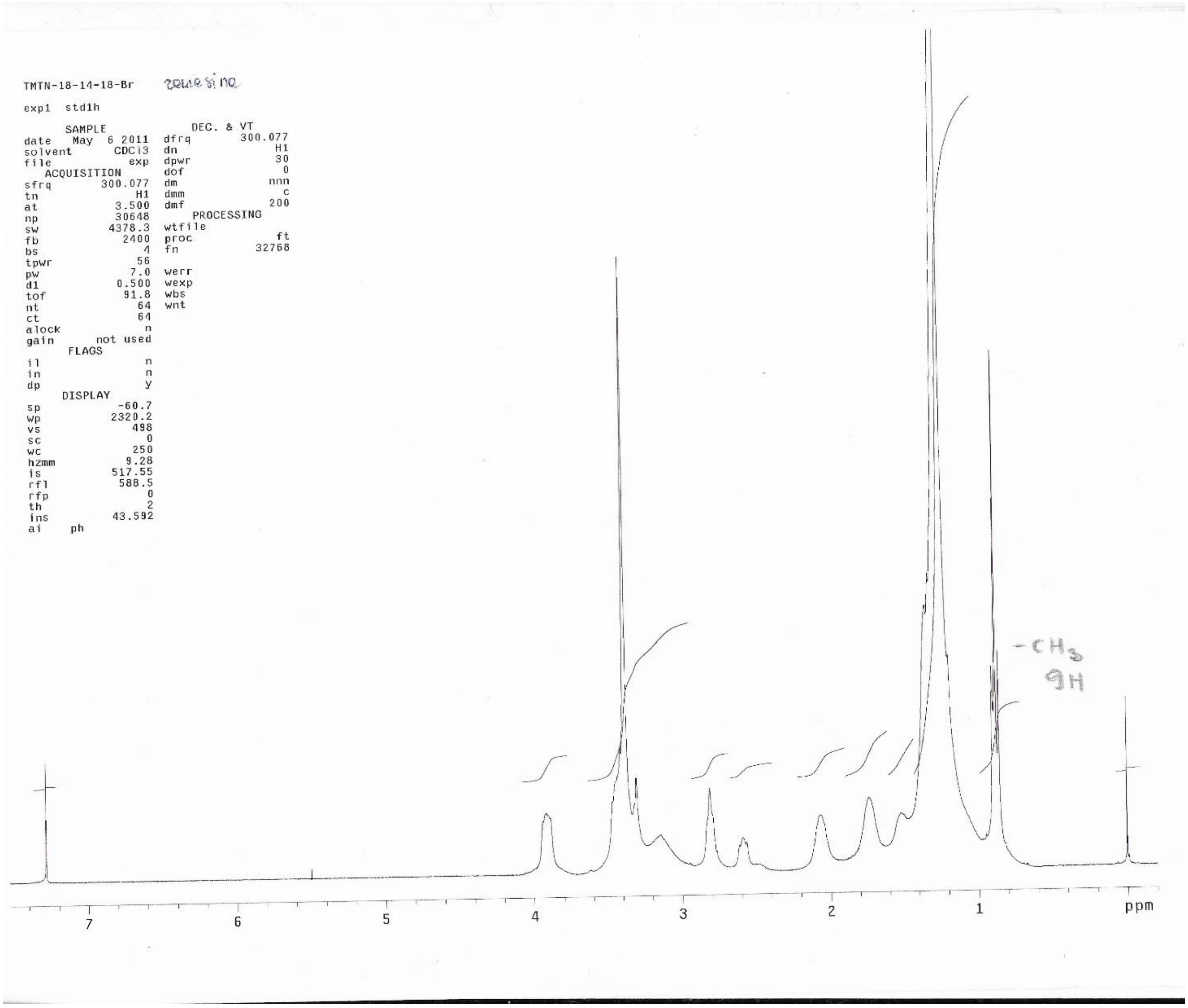

Compound 15 ^{13}C NMR

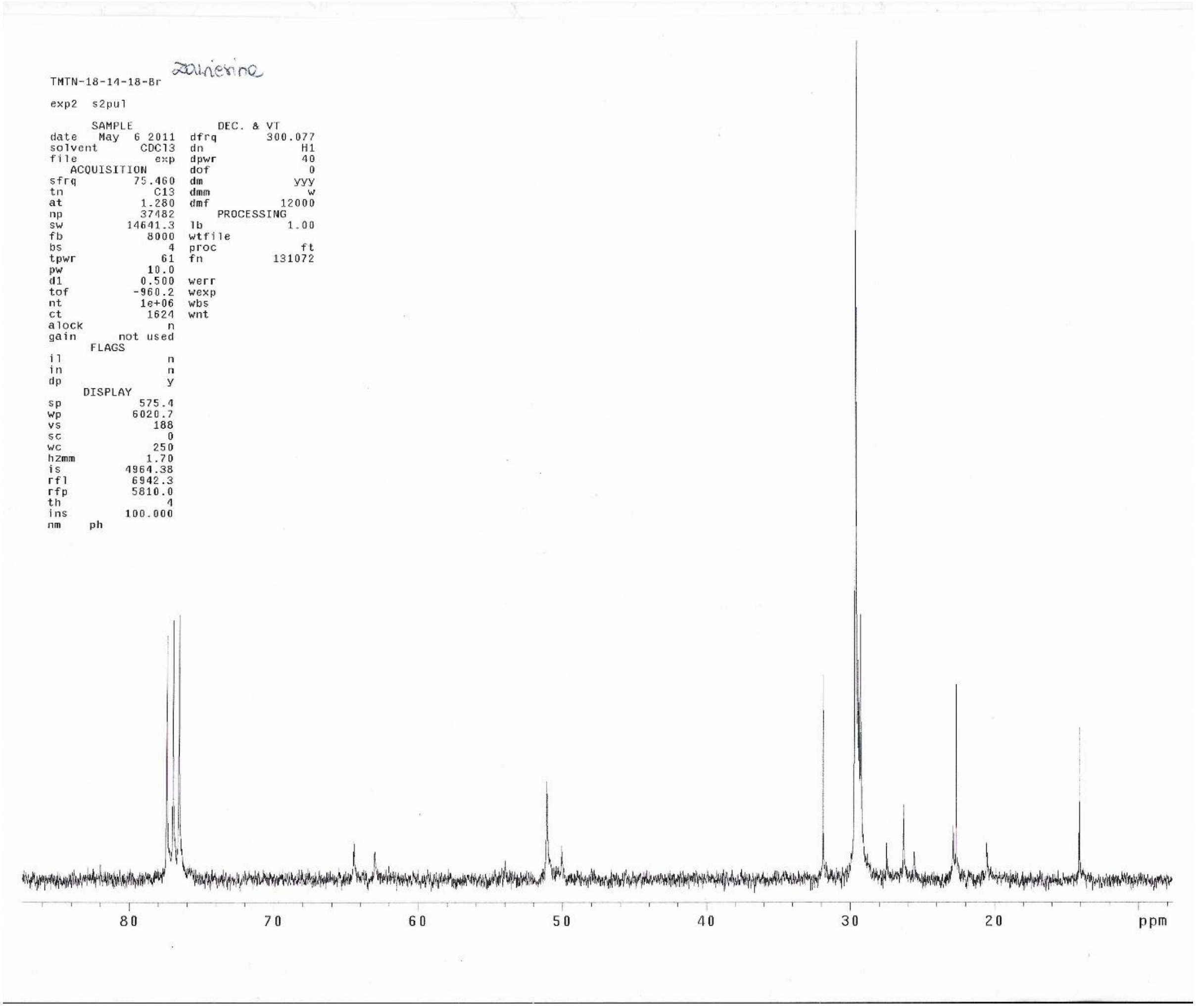

Compound 16 ^{1}H NMR

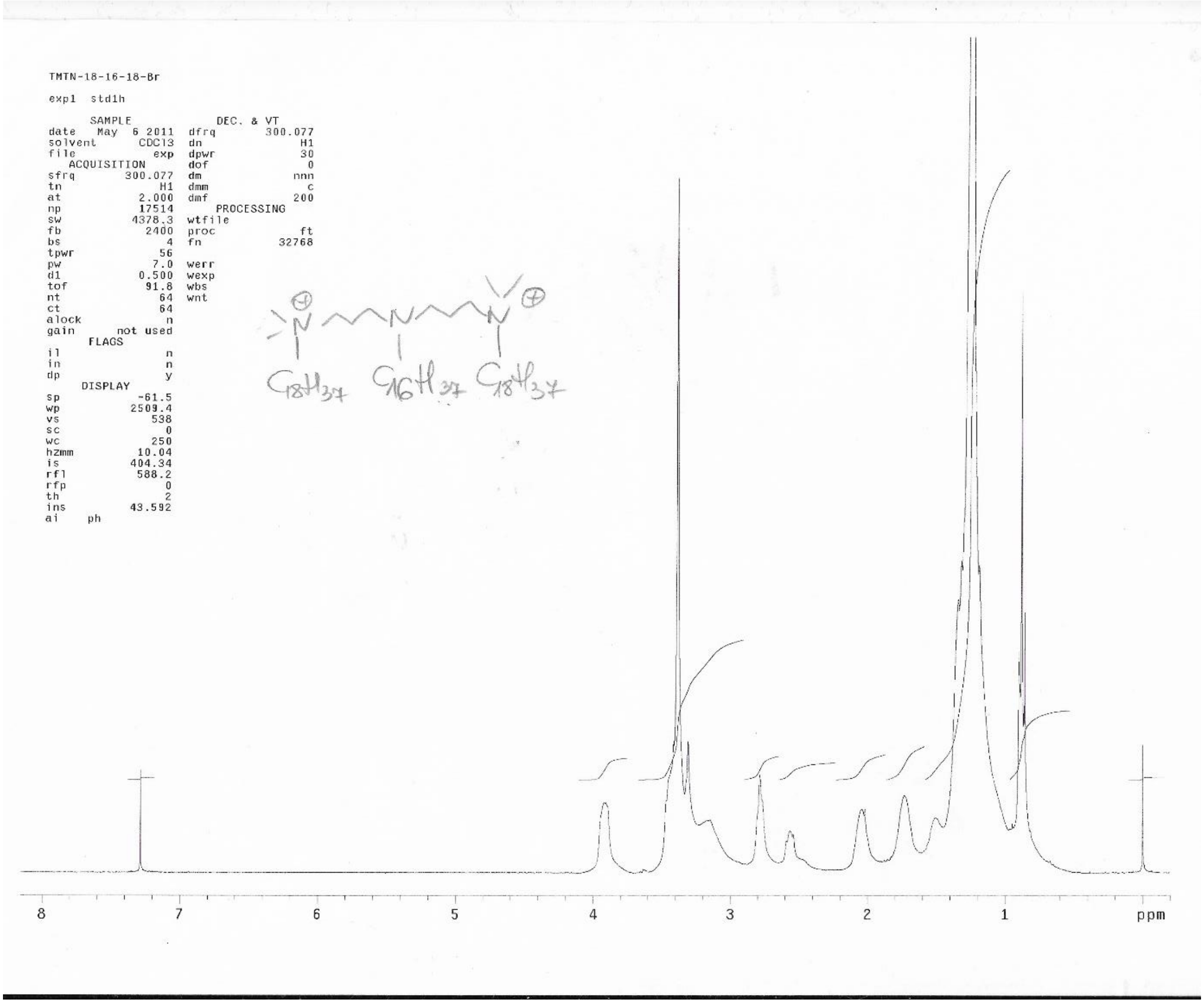

Compound 16 ^{13}C NMR

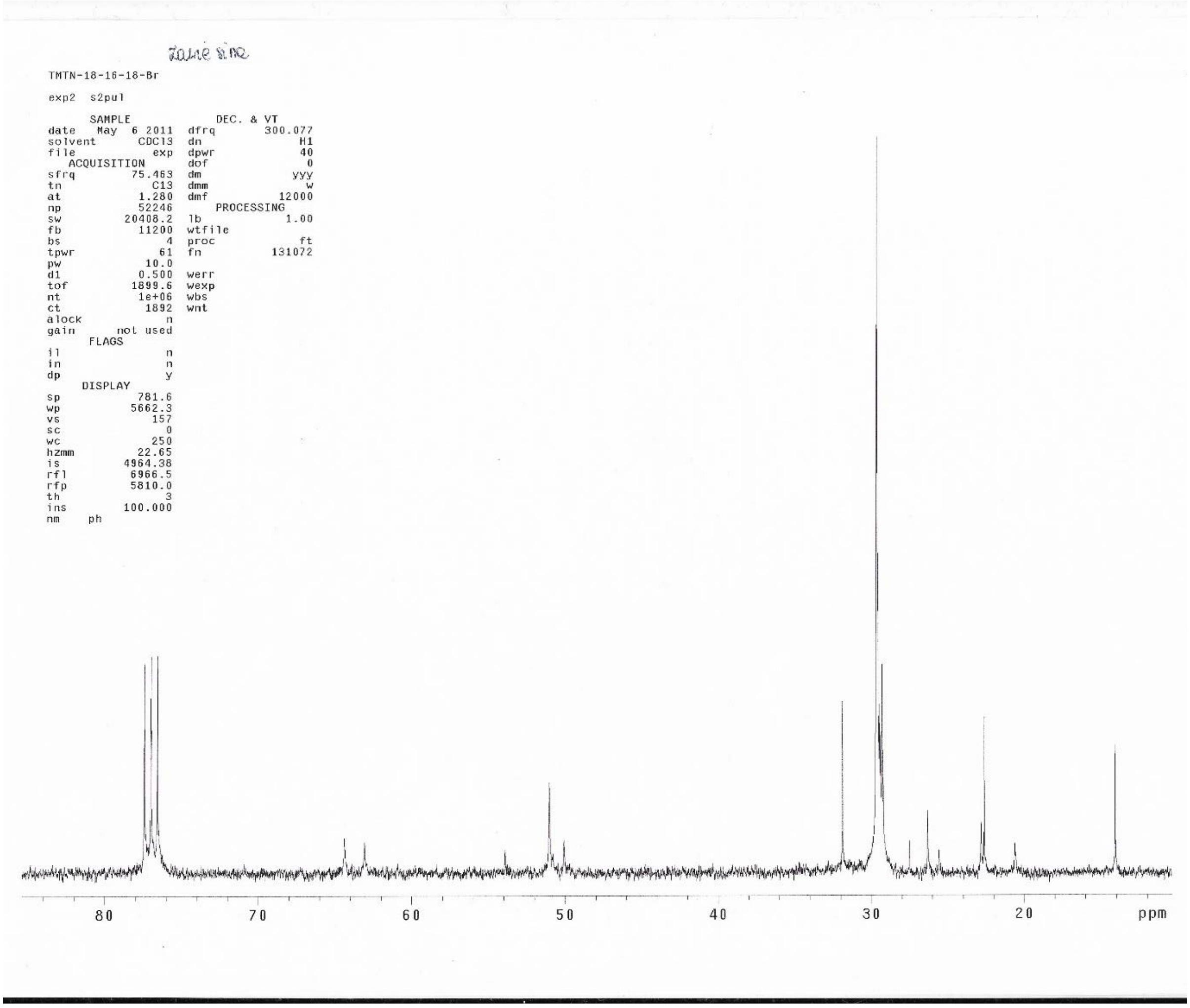

Compound 17 ^{1}H NMR

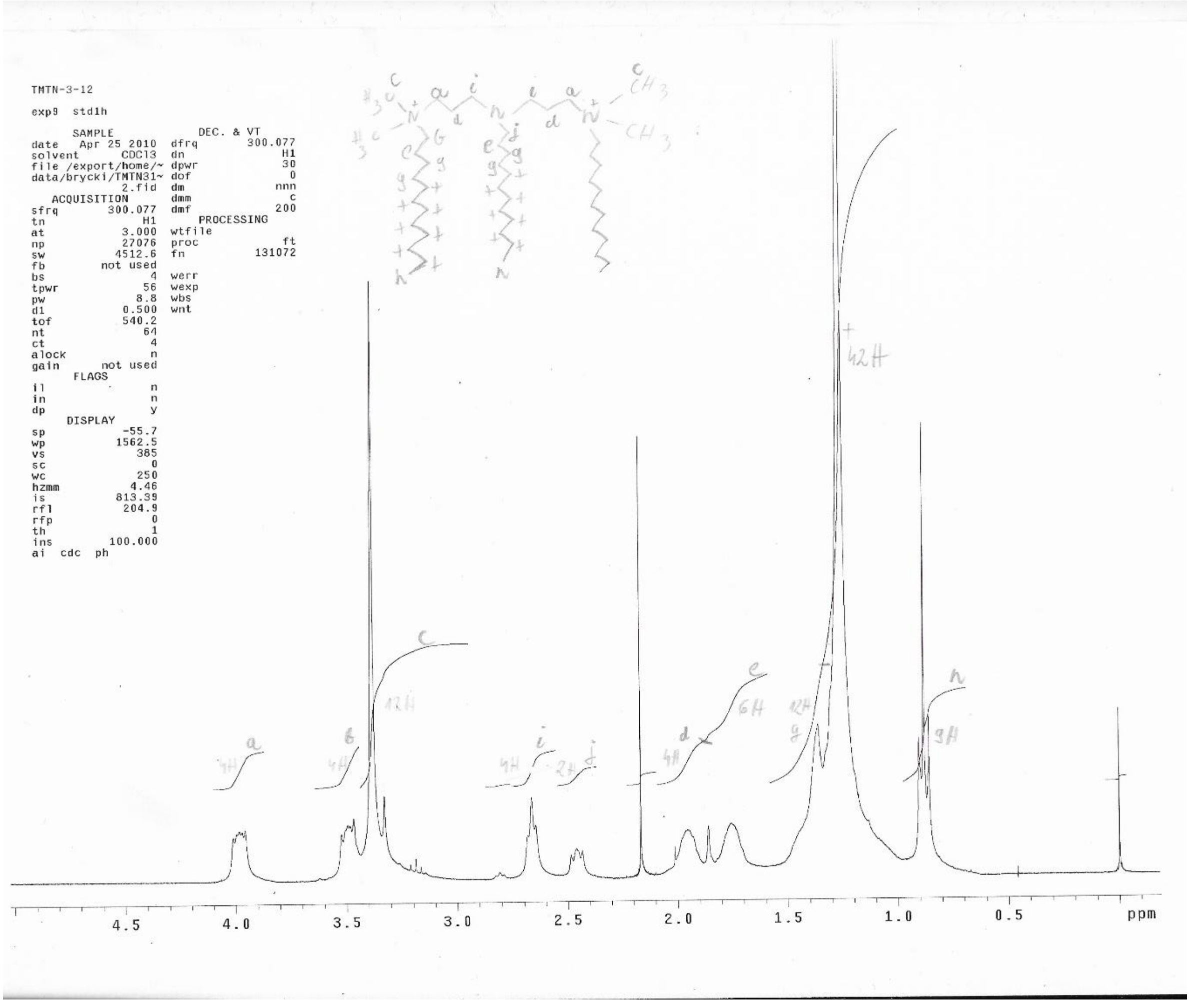

Compound 17 ^{13}C NMR

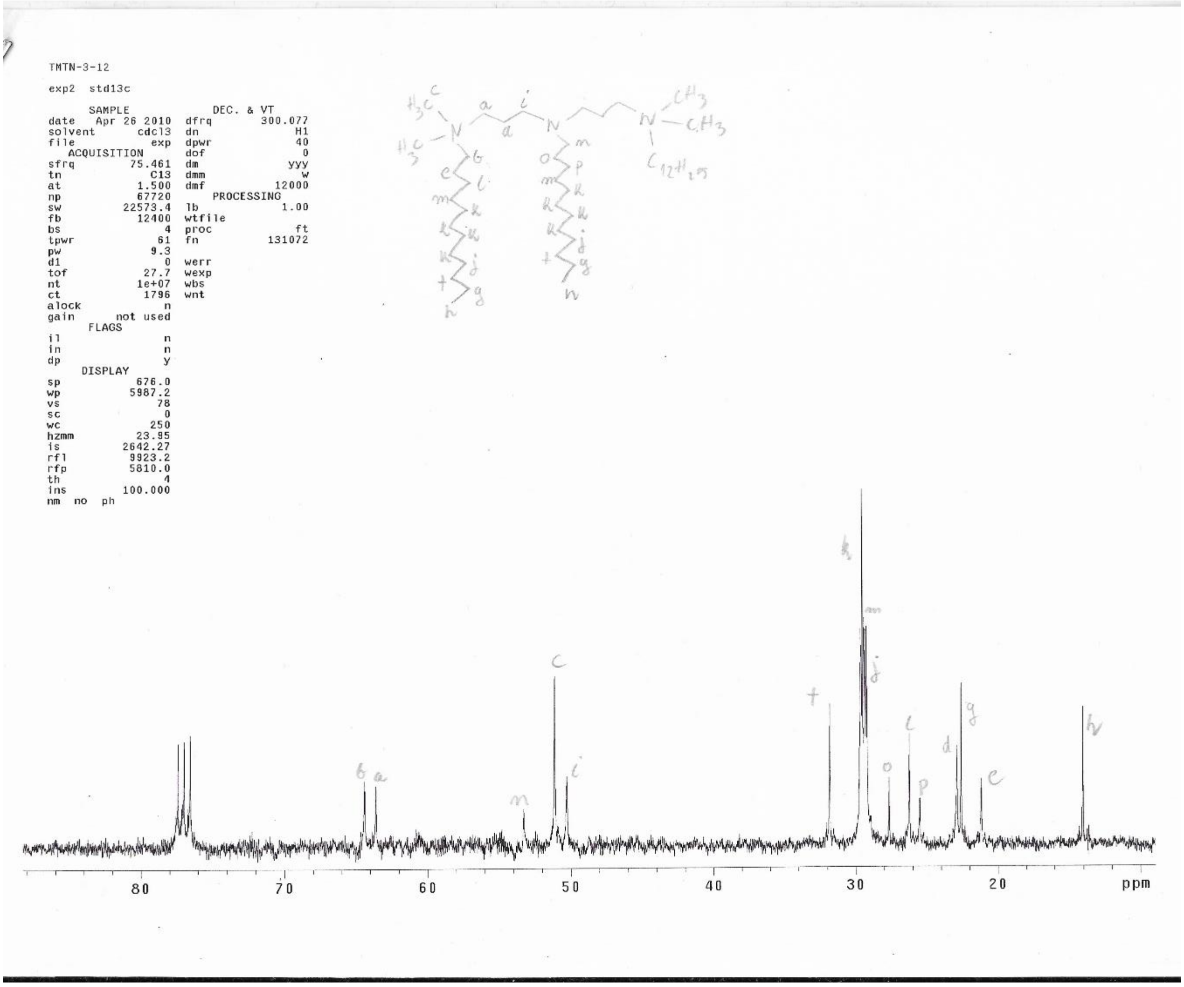

Compound 18 ^{1}H NMR

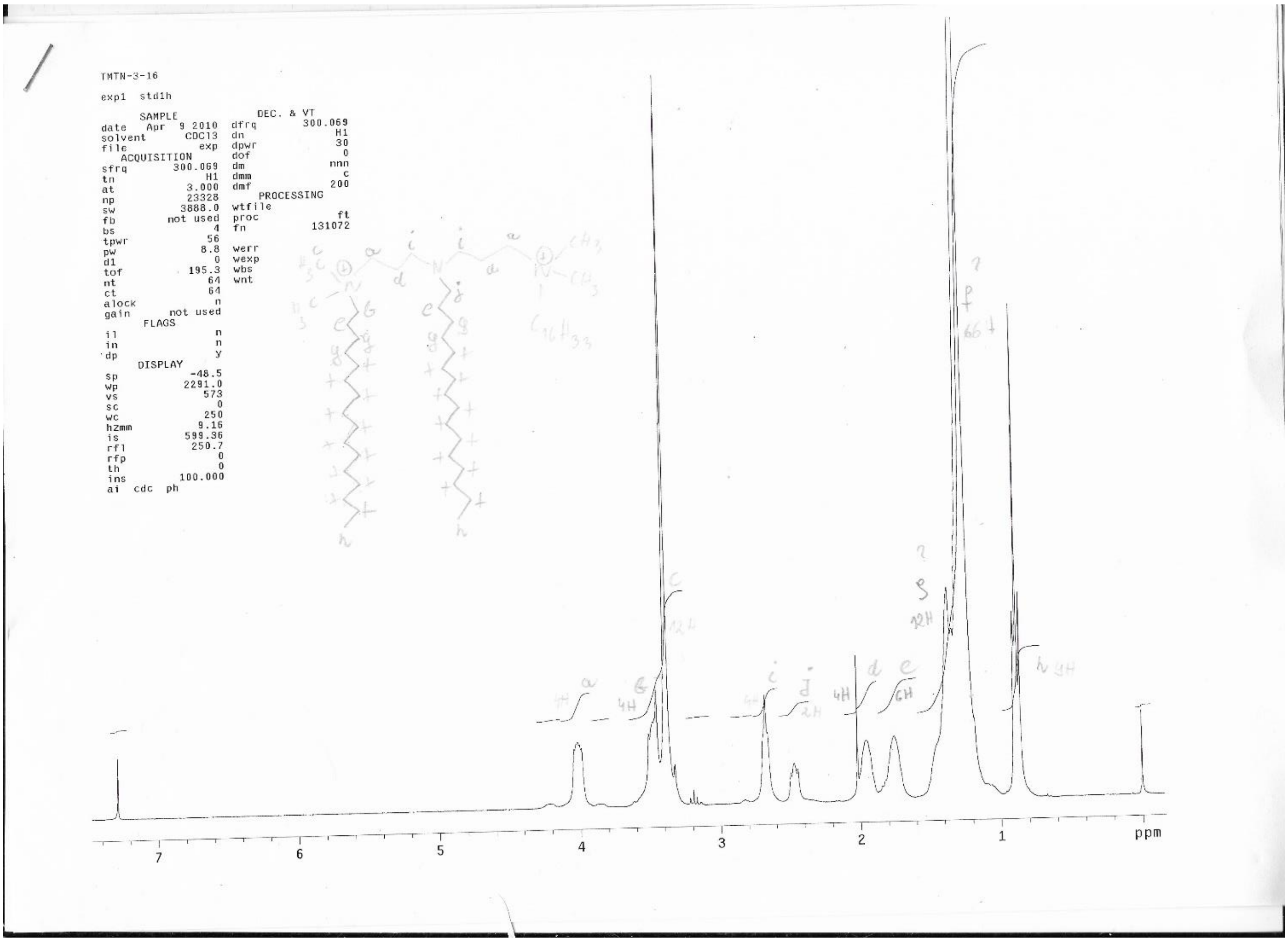

Compound 18 ^{13}C NMR

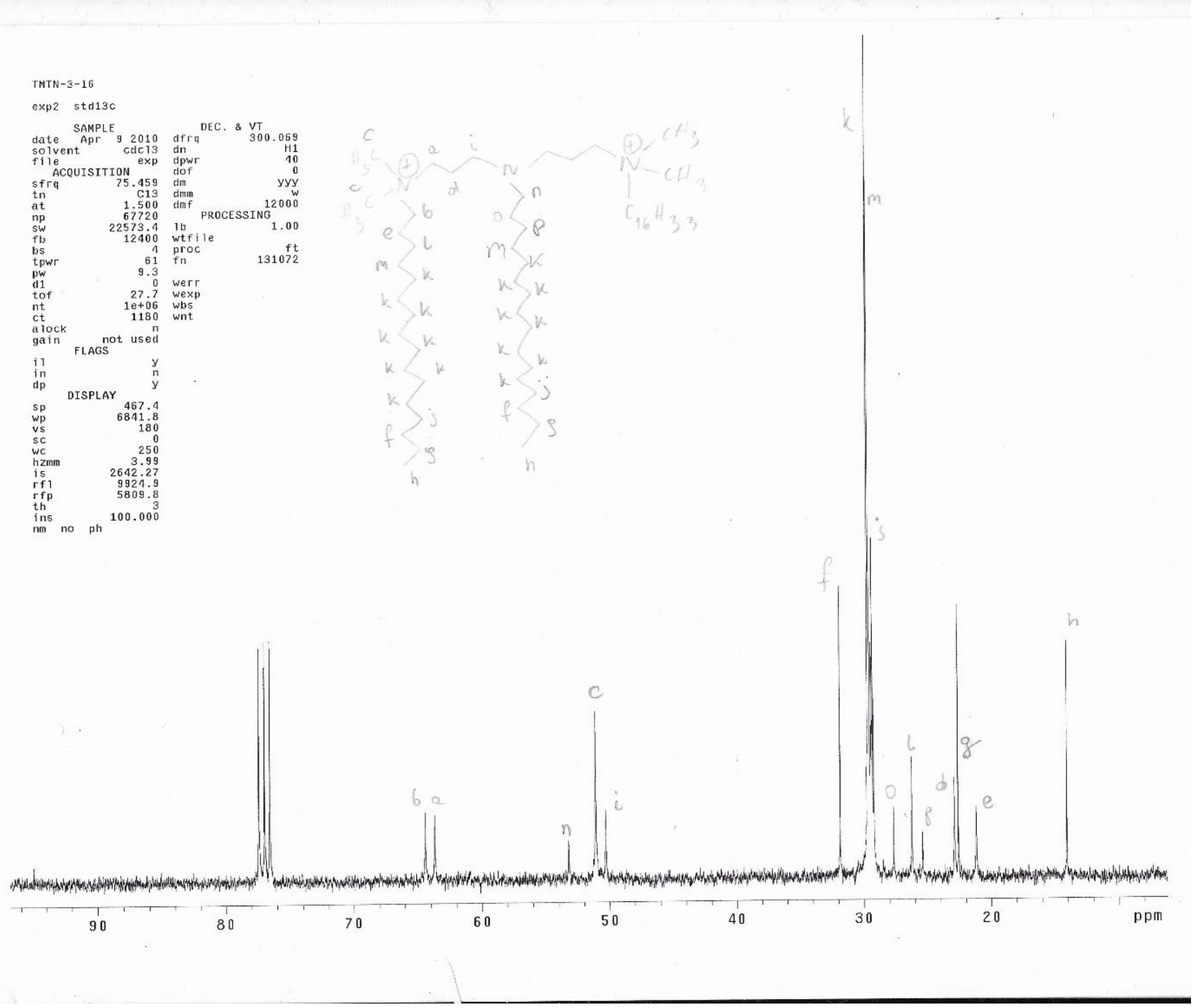

Compound 19 ^{1}H NMR

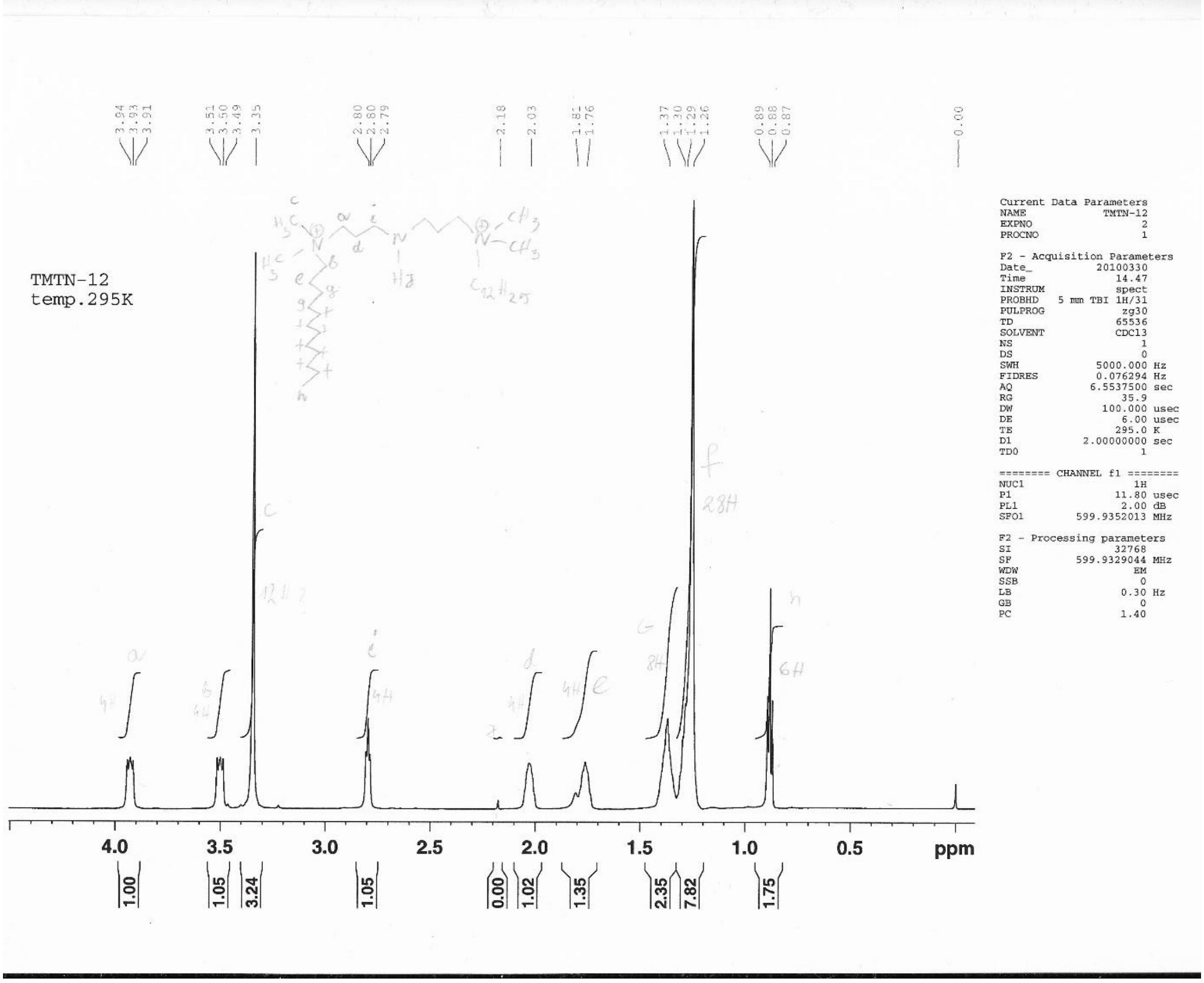

Compound 19 ^{13}C NMR

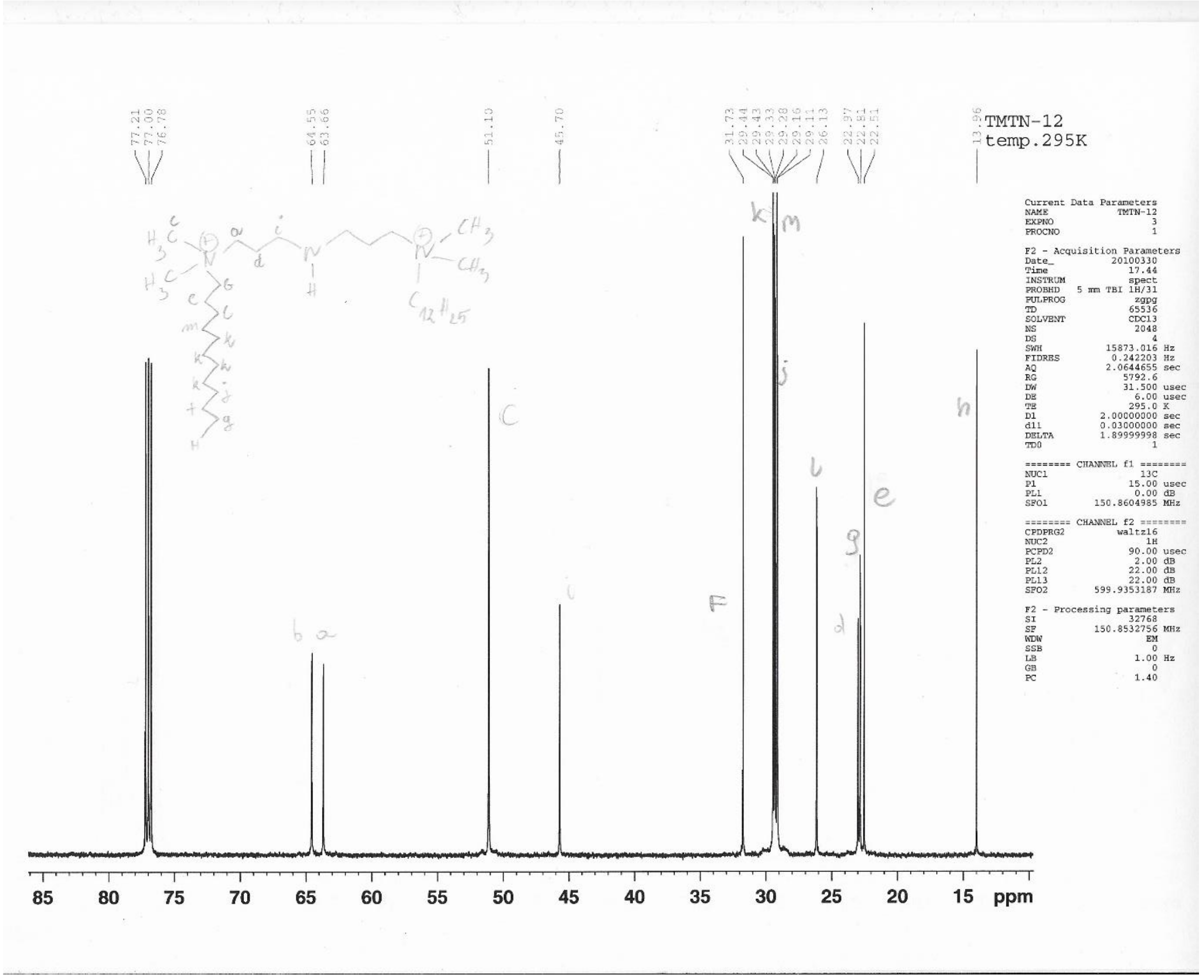

Compound 20 ^{1}H NMR

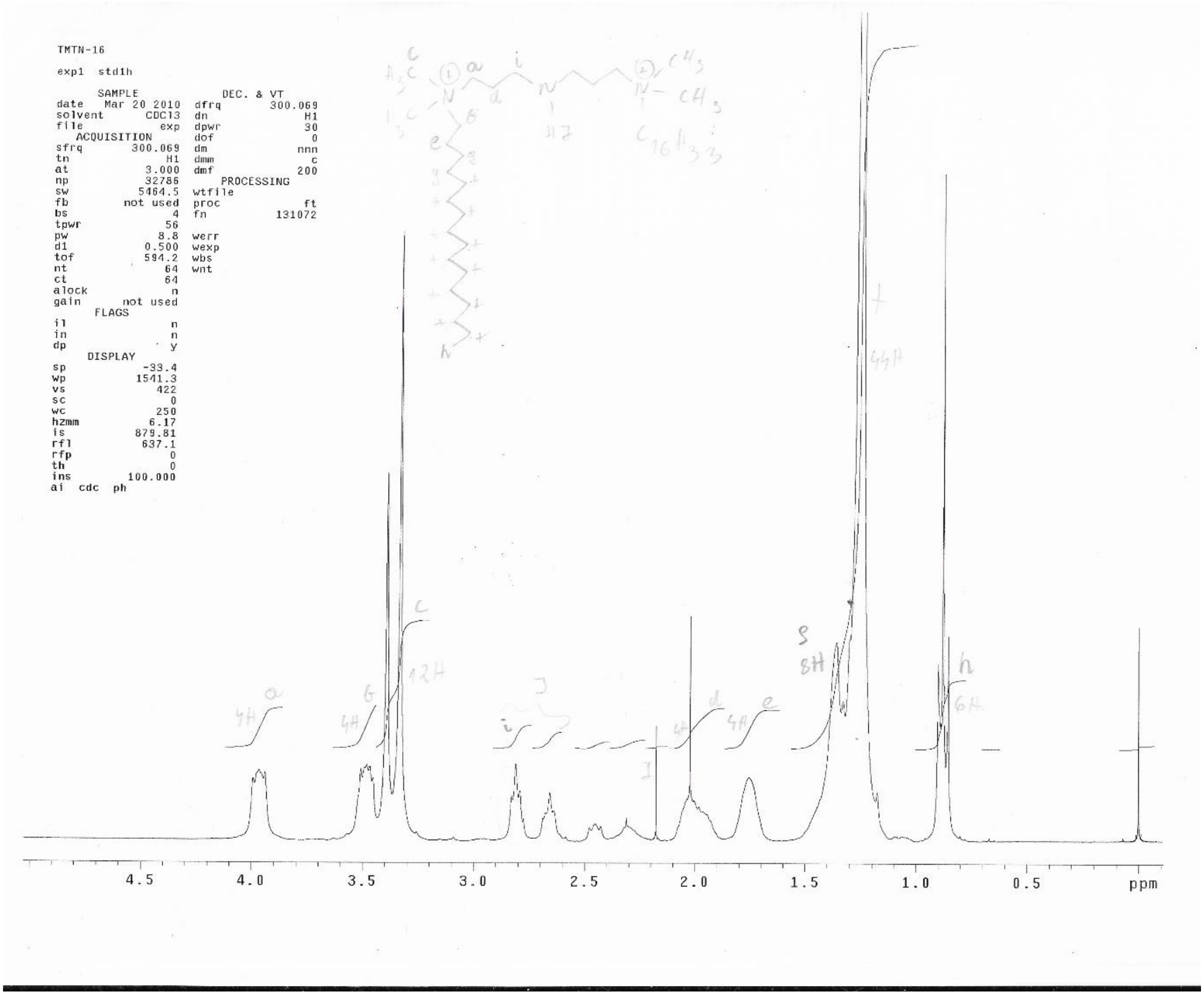

Compound 20 ^{13}C NMR

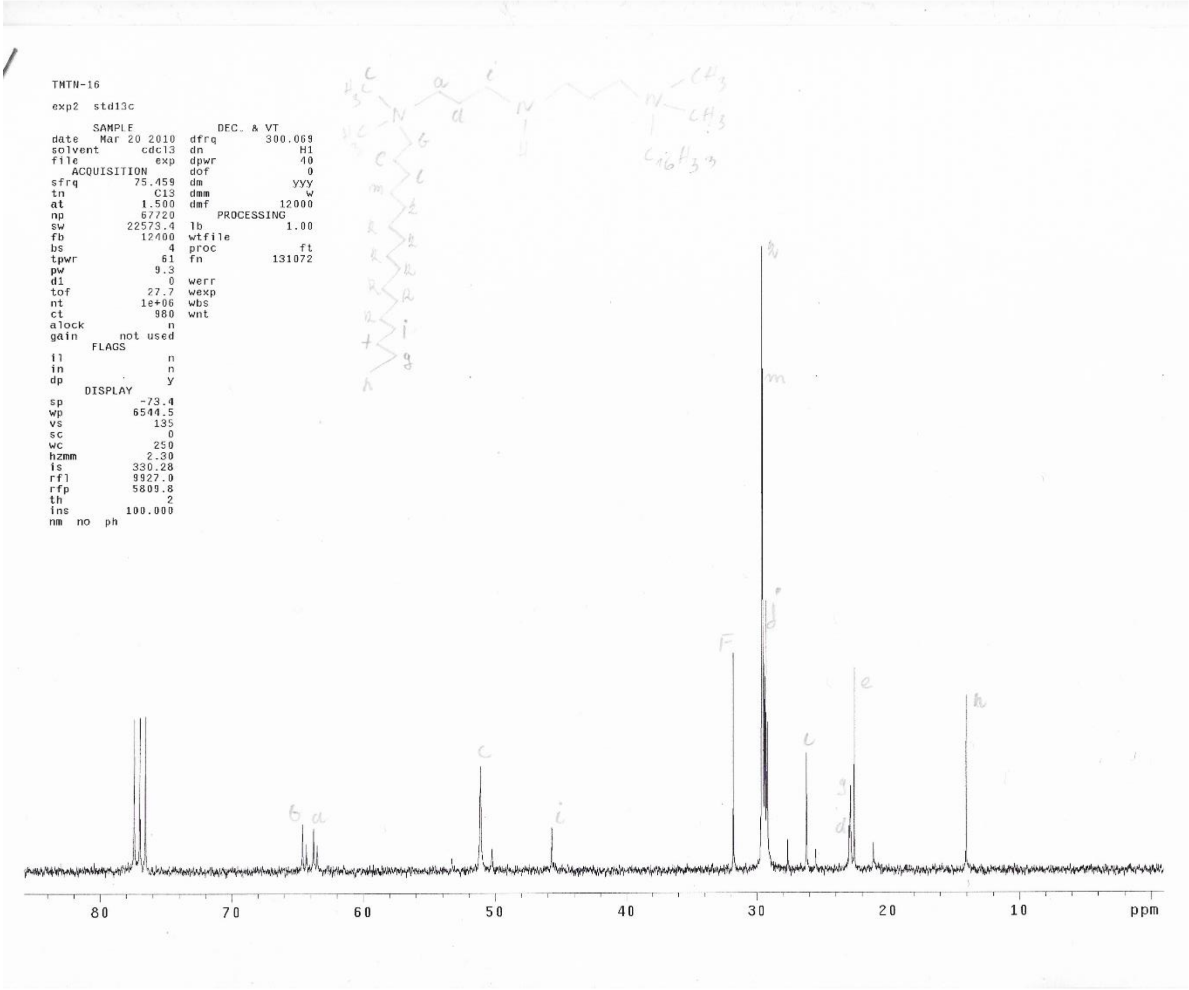

Compound 21 ^1H NMR

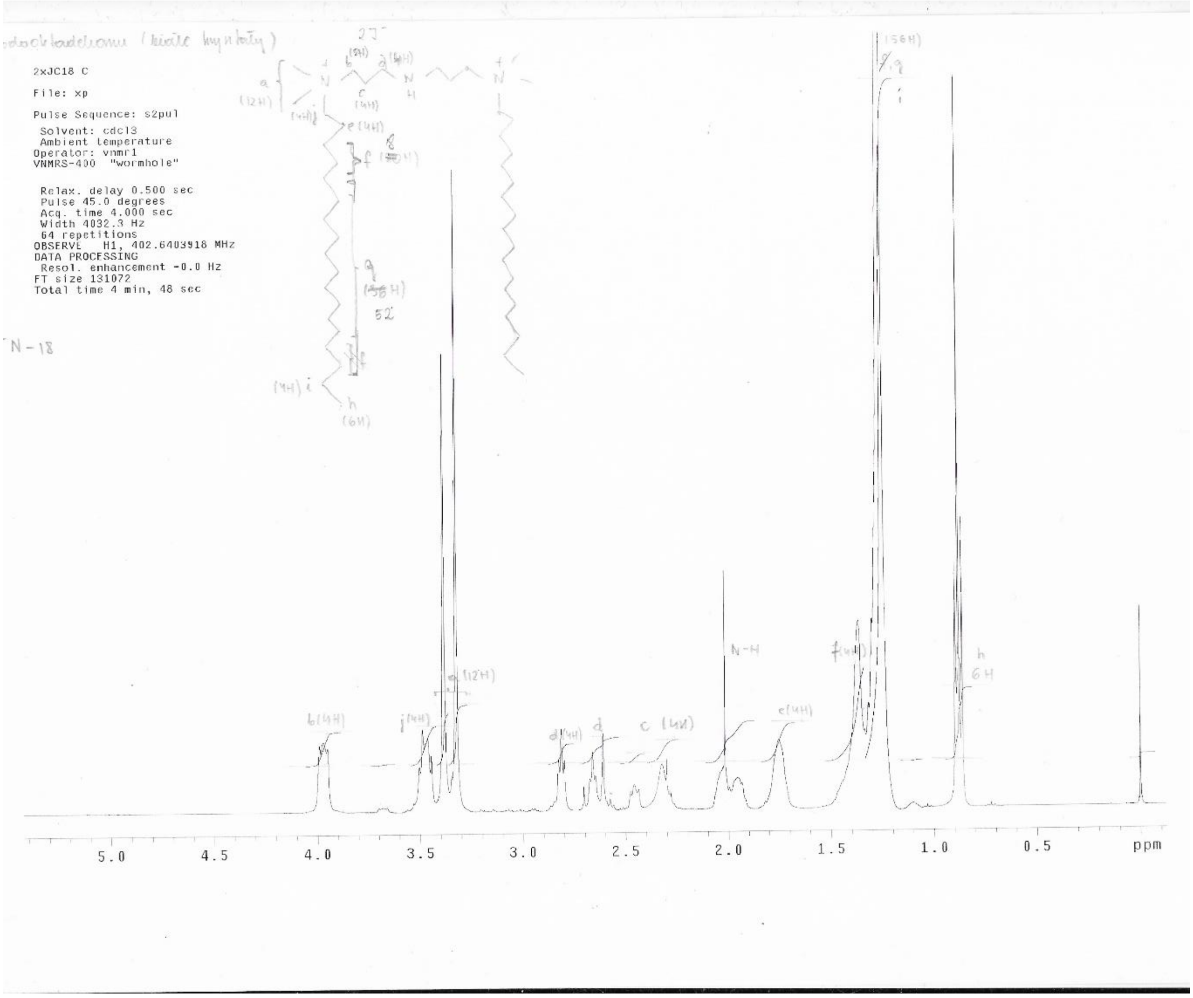

Compound 21 ^{13}C NMR

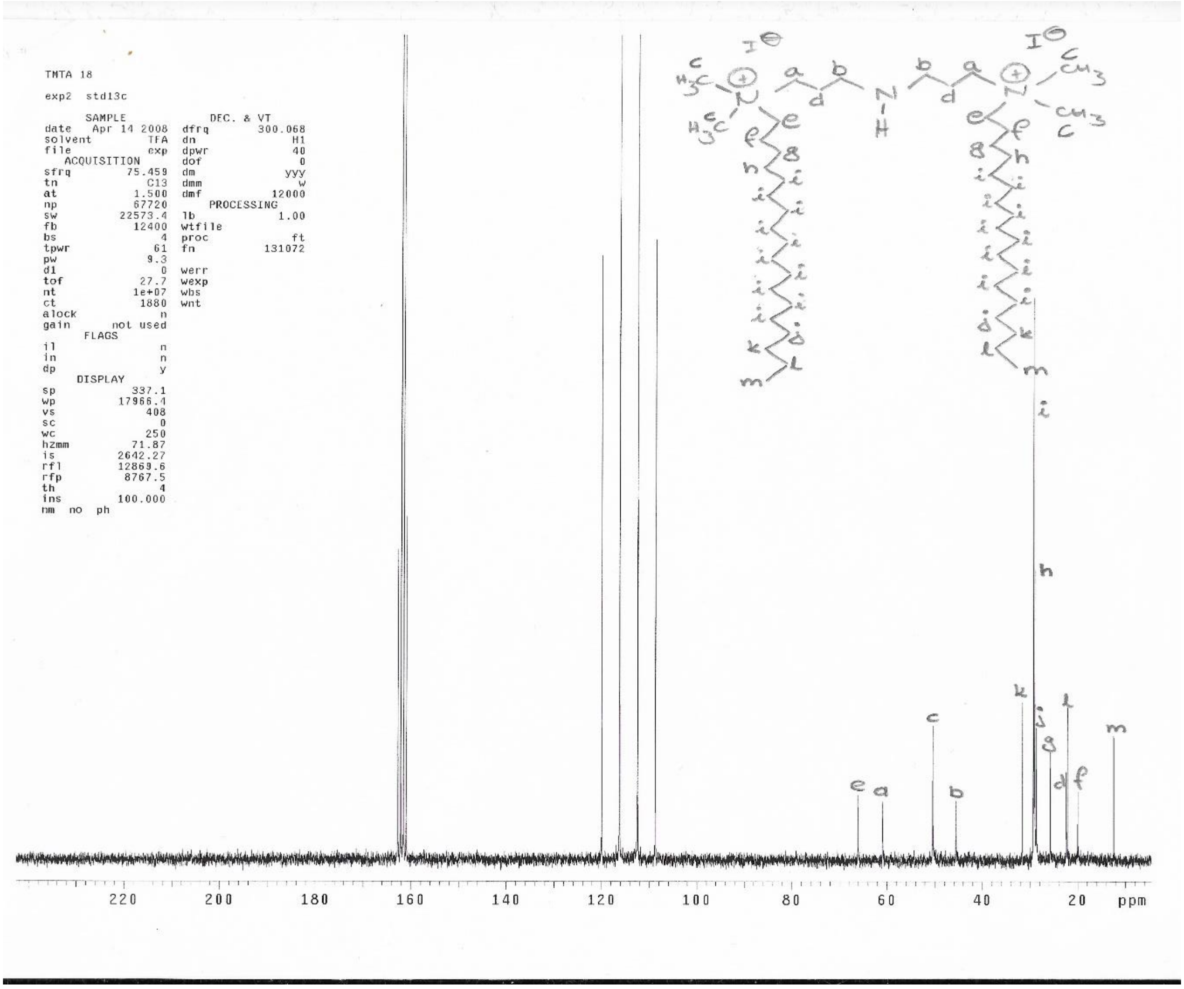

Supplement: Supplementary file 1 [file molecules-25-04054-s001.pdf]
